# microRNA-132 regulates gene expression programs involved in microglial homeostasis

**DOI:** 10.1016/j.isci.2023.106829

**Published:** 2023-05-06

**Authors:** Hannah Walgrave, Amber Penning, Giorgia Tosoni, Sarah Snoeck, Kristofer Davie, Emma Davis, Leen Wolfs, Annerieke Sierksma, Mayte Mars, Taofeng Bu, Nicola Thrupp, Lujia Zhou, Diederik Moechars, Renzo Mancuso, Mark Fiers, Andrew J.M. Howden, Bart De Strooper, Evgenia Salta

**Affiliations:** 1 VIB-KU Leuven Center for Brain & Disease Research, 3000 Leuven, Belgium; 2 KU Leuven, Department of Neurosciences, Leuven Brain Institute (LBI), 3000 Leuven, Belgium; 3 Netherlands Institute for Neuroscience, 1105 BA Amsterdam, the Netherlands; 4 VIB-KU Leuven Center for Brain & Disease Research, Bioinformatics Core Facility, 3000 Leuven, Belgium; 5 UK Dementia Research Institute at UCL, London WC1E 6BT, UK; 6 Discovery Neuroscience, Janssen Research and Development, Division of Janssen Pharmaceutica NV, 2340 Beerse, Belgium; 7 Microglia and Inflammation in Neurological Disorders (MIND) Lab, VIB Center for Molecular Neurology, VIB, 2610 Antwerp, Belgium; 8 Department of Biomedical Sciences, University of Antwerp, 2610 Antwerp, Belgium; 9 UK Dementia Research Institute, University of Dundee, Dundee DD1 4HN, UK

**Keywords:** Molecular biology, Neuroscience, Omics

## Abstract

microRNA-132 (miR-132), a known neuronal regulator, is one of the most robustly downregulated microRNAs (miRNAs) in the brain of Alzheimer’s disease (AD) patients. Increasing miR-132 in AD mouse brain ameliorates amyloid and Tau pathologies, and also restores adult hippocampal neurogenesis and memory deficits. However, the functional pleiotropy of miRNAs requires in-depth analysis of the effects of miR-132 supplementation before it can be moved forward for AD therapy. We employ here miR-132 loss- and gain-of-function approaches using single-cell transcriptomics, proteomics, and *in silico* AGO-CLIP datasets to identify molecular pathways targeted by miR-132 in mouse hippocampus. We find that miR-132 modulation significantly affects the transition of microglia from a disease-associated to a homeostatic cell state. We confirm the regulatory role of miR-132 in shifting microglial cell states using human microglial cultures derived from induced pluripotent stem cells.

## Highlights

• MiR-132 is downregulated in Alzheimer’s disease (AD) brain
• Mir-132, mainly known for neuronal functions, regulates many aspects of AD pathology
• MiR-132 manipulation in mouse hippocampus reveals a role in microglial homeostasis
• MiR-132 shifts disease-associated to homeostatic microglia in iPSC-derived cultures

## Graphical abstract

**Figure:**
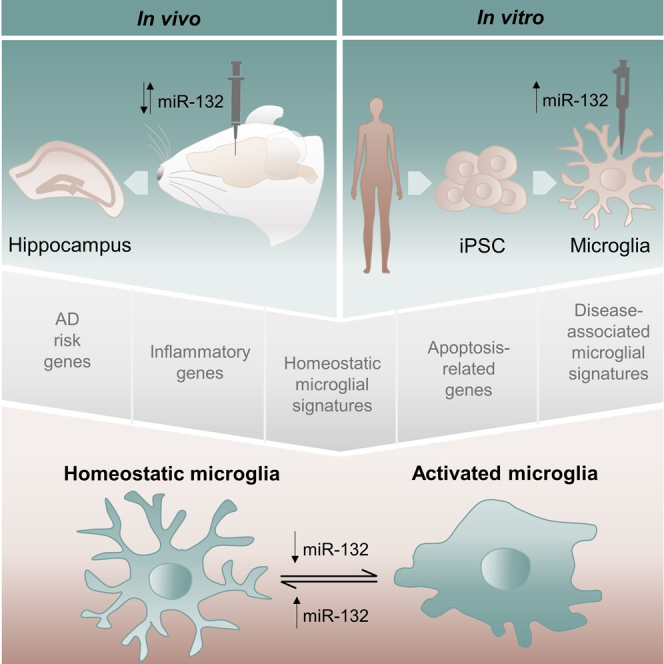

## Introduction

miRNAs are endogenous small noncoding RNAs that primarily operate as part of the cellular gene silencing machinery. By binding complementary sequences in target messenger RNAs (mRNAs), miRNAs inhibit translation or induce mRNA degradation.^1^^,^^2^^,^^3^ miRNAs can concomitantly regulate numerous targets and are, therefore, thought to mainly function as “fine-tuners” of core cellular functions, such as maintaining tissue homeostasis in the presence of stressors.^4^^,^^5^ In the central nervous system (CNS), miRNAs control gene expression in diverse cellular populations.^6^^,^^7^^,^^8^^,^^9^^,^^10^^,^^11^ The functional pleiotropy of miRNAs renders them, in principle, interesting candidates to treat complex multifactorial diseases, such as AD, as they can potentially “hit” multiple sensitive nodes of a pathogenic molecular cascade.^12^^,^^13^^,^^14^^,^^15^^,^^16^

miRNA profiles are altered in the brain of AD patients, often from early disease stages onwards.^17^^,^^18^^,^^19^^,^^20^^,^^21^^,^^22^ Several of these deregulated miRNAs directly regulate key molecular processes in AD pathogenesis including amyloidosis, Tau pathology, (neuro)inflammation, neuronal death, and memory impairment.^16^ miR-132, one of the most abundant, brain-enriched miRNAs, and the most consistently downregulated miRNA in human AD brain, exemplifies the multi-targeting potential of miRNAs in the context of AD.^16^^,^^20^^,^^23^^,^^24^ More specifically, miR-132 can ameliorate AD pathology by regulating a series of targets involved in amyloid plaque accumulation, Tau hyperphosphorylation and metabolism, neuronal survival, neurotrophic signaling, adult neurogenesis, and memory formation.^23^^,^^24^^,^^25^^,^^26^^,^^27^^,^^28^^,^^29^^,^^30^^,^^31^^,^^32^^,^^33^^,^^34^^,^^35^^,^^36^^,^^37^^,^^38^ miR-132 expression variation could explain 6.7% of the observed variance in AD histopathology and mediate part of the effect of the AD polygenic risk score on global cognitive decline.^39^^,^^40^ miR-132 supplementation in AD mouse models ameliorates amyloid and Tau pathology and improves memory performance.^23^^,^^26^^,^^32^^,^^33^^,^^35^ Although these findings are indicative of the therapeutic potential of miR-132 in AD, it remains unclear how altering miR-132 in the brain would broadly impact molecular networks and biological cascades across different cell types.

Identifying molecular targets of any miRNA remains a significant challenge. While the primary effects of miRNAs involve inhibiting the translation of mRNAs, a large part of miRNA regulation results in alterations of the transcriptome.^2^^,^^3^^,^^41^^,^^42^^,^^43^^,^^44^^,^^45^ Moreover, miRNAs exert modest effects on individual targets. It is assumed that the accumulation of such subtle changes in the expression of individual genes in a specific molecular pathway might explain the overall biological consequences of miRNA regulation.^23^^,^^32^^,^^35^^,^^46^^,^^47^^,^^48^^,^^49^^,^^50^^,^^51^ The regulatory impact of a given miRNA can dramatically vary across individual cells.^52^^,^^53^^,^^54^^,^^55^^,^^56^^,^^57^ Systematic profiling of cell type-specific miRNA-mRNA interactions using orthogonal approaches may, therefore, reveal putative selective regulatory effects.^58^^,^^59^

Here, we hypothesized that the combinatorial contribution of proteomic and transcriptomic regulatory effects of miR-132 converges onto specific biological pathways in distinct cellular populations. Overall, detectable effects on the proteome and on individual putative miR-132 targets turned out to be subtle. Yet, miR-132 overexpression and knockdown in the hippocampus resulted in a consistent shift across microglial cell states. We confirm these observations in human induced pluripotent stem cell (iPSC)-derived microglial cultures, where miR-132 overexpression promoted the expression of homeostatic transcriptional programs, while repressing disease-associated activation signatures. Further work is needed to elucidate whether regulation of microglial subpopulations by miR-132 could have beneficial or detrimental implications in the context of AD.

## Results

### *In silico* identification of putative miR-132 targets in the brain

**Figure 1:**
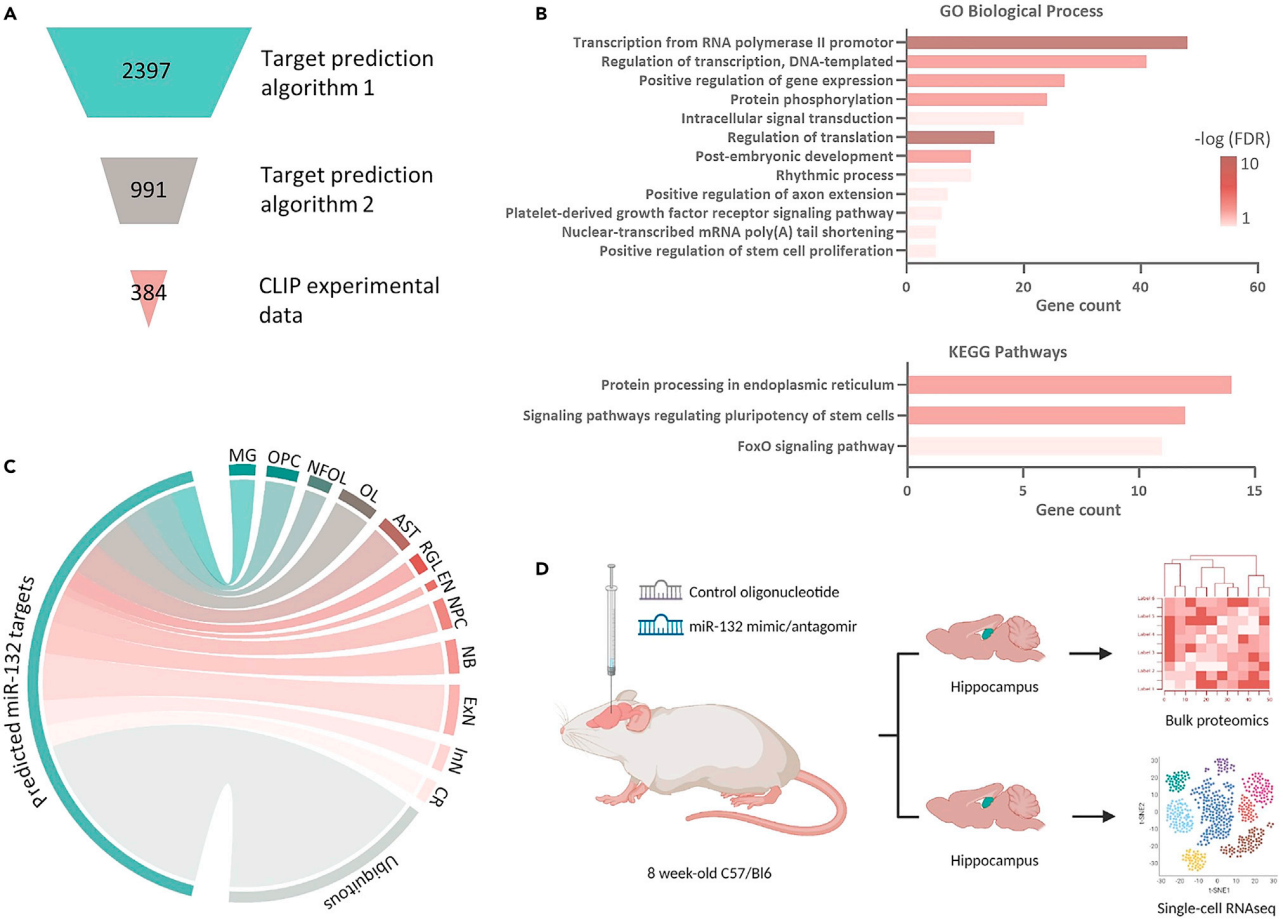
Identification and possible function of miR-132 predicted targets in brain (A) Schematic representation of the identification strategy of predicted miR-132 targets, using an intersection of two target prediction algorithms and a high-confidence threshold for experimental data from AGO-CLIP-seq experiments.^62^ (B) All significantly enriched GO biological processes and KEGG pathways for 384 predicted miR-132 targets. Color indicates significance (Fisher’s Exact test, p values corrected with false discovery rate (FDR), adjusted p value <0.05 considered significant). (C) Circos plot depicting cell type-specific expression profiles of predicted miR-132 targets. (D) Schematic representation of experimental outline. MG, Microglia; OPC, oligodendrocyte precursor cell; NFOL, newly formed oligodendrocyte; OL, oligodendrocyte; AST, astrocyte; RGL, radial-glia like cell; EN, endothelia; NPC, neuronal precursor cell; NB, neuroblast; ExN, excitatory neuron; InN, inhibitory/GABAergic neuron; CR, Cajal-Retzius neuron. See also Table S1.

Predicted mRNA targets of highly tissue-specific miRNAs are generally expressed in the same tissue as the miRNA.^60^^,^^61^ We initially employed an *in silico* approach to extract predicted miR-132 targets and characterize their expression in diverse cellular populations in the brain. We made use of the Encori database,^62^ which provides the possibility to explore several target prediction algorithms together with AGO crosslinking immunoprecipitation sequencing (CLIP-seq) data^58^^,^^63^ to identify targets of any microRNA of interest. We selected 384 predicted miR-132 targets based on the combination of at least 2 target prediction algorithms and a high-confidence threshold for AGO-CLIP-seq (Figure 1A and Table S1). To functionally annotate the selected miR-132 predicted targets, we used the database for annotation, visualization and integrated discovery (DAVID)^64^^,^^65^ and identified significantly enriched [Fisher’s Exact test, false discovery rate (FDR)-adjusted p value <0.05] gene ontology (GO) biological processes and Kyoto encyclopedia of genes and genomes (KEGG) pathways. Not unexpectedly, regulation of transcription and translation and other rather generic functional categories known to be regulated by miRNAs,^42^^,^^52^^,^^66^^,^^67^ were depicted. In addition, previously reported miR-132-regulated processes, such as stem cell pluripotency and proliferation,^28^^,^^29^^,^^35^ axon extension,^34^^,^^36^^,^^68^^,^^69^ and cellular homeostasis, autophagy and apoptosis, including FOXO signaling,^20^^,^^38^^,^^70^^,^^71^ were identified among the significantly enriched pathways (Figure 1B).

We next asked in which brain cell types the *in silico* predicted targets of miR-132 are expressed. We assessed cell type-specific expression using a single-cell RNA sequencing (scRNAseq) dataset from mouse hippocampus.^72^ Predicted miR-132 targets were expressed in neurons, oligodendrocytes, microglia, and astrocytes (Figure 1C and Table S1), suggesting that miR-132 itself may also be similarly expressed in various cell types. We confirmed expression of miR-132 in oligodendrocytes, microglia, astrocytes, and neurons isolated from mouse hippocampus using magnetic activated cell sorting (MACS) and fluorescence-activated cell sorting (FACS) (Figures S1A, S1D, S1G, S1H, and S1J). Taken together, these data suggest a putative functional role for miR-132 in different cellular populations in the brain. To test this hypothesis, we set out to experimentally explore miR-132-regulated cellular targets in mouse hippocampus using a combinatorial proteomics and single-cell transcriptomics approach (Figure 1D).

### miR-132 depletion affects divergent biological pathways in different cellular populations of the hippocampus but effects on individual targets are small

To explore physiologically relevant effects of miR-132 regulation we first employed a loss-of-function approach. More specifically, to determine the impact of miR-132 knockdown in the hippocampus, we intracerebroventricularly (ICV) infused wild-type mice with antisense (miR-132 antagomiR, miR-132 KD) or corresponding control (Control) oligonucleotides (Figure S1B) and assessed the effects on the proteome in bulk and on the transcriptome at single-cell resolution. The expression levels of miR-212, a cognate miRNA, which is co-transcribed with miR-132 and shares a similar mature sequence, did not change upon miR-132 KD (Figure S1I). While miR-132 knockdown was consistent in isolated hippocampal oligodendrocytes, the decrease was less robust in astrocytes and microglia, whereas, unexpectedly, no reduction in miR-132 levels was detected in neurons, precluding the interpretation of any observed functional alterations in neuronal clusters (Figure S1E).

**Figure 2:**
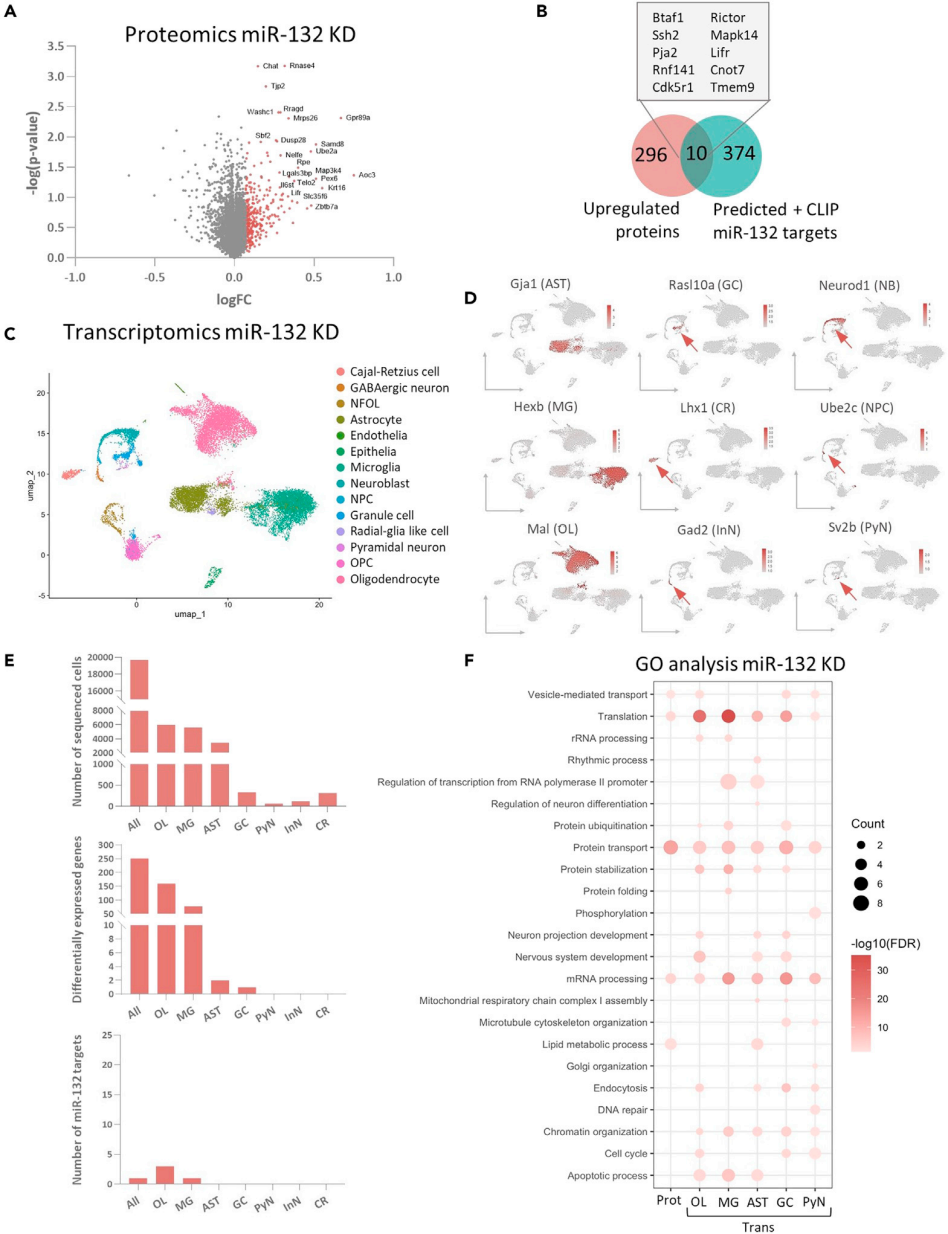
Impact of miR-132 depletion in the hippocampus (A) Differentially expressed proteins upon miR-132 KD presented in a volcano plot. The top 5% of proteins anticorrelated to miR-132 are indicated in red. (B) Identification of putative miR-132 targets from intersection with 5% most upregulated proteins and the list of identified predicted miR-132 targets. (C) UMAP visualization of 19,705 isolated mouse hippocampal miR-132 KD and corresponding control single-cell transcriptomes. Cells are colored by identified cell type. NFOL, newly formed oligodendrocyte; NPC, neuronal precursor cell; OPC, oligodendrocyte precursor cell. (D) UMAP plots colored by the normalized expression level of cell type-specific marker genes used for cluster annotation. Arrows indicate the cell cluster of interest. (E) Number of cells, number of significant DEGs (Wilcoxon rank-sum test using Bonferroni for p value correction, adjusted p value <0.05 considered significant), or amount of putative miR-132 targets identified by intersection with predicted miR-132 targets, as included in the scRNAseq analysis. Counts correspond to all cells pseudo-bulked together (All) or to each cell type. (F) GO biological processes significantly enriched in differentially expressed proteins (Prot) or genes (Trans) in distinct cell types. Count represents the % of included proteins/genes that are part of each process. Color represents significance, top 20 GO terms are displayed (Fisher’s Exact test, p values corrected with FDR, adjusted p value <0.05 considered significant). AST, astrocyte; GC, granule cell; NB, neuroblast; MG, microglia; CR, Cajal-Retzius neuron; NPC, neuronal precursor cell; OL, oligodendrocyte; InN, inhibitory/GABAergic neuron; PyN, hippocampal pyramidal neuron. See also Figures S1, S2, Tables S2, and S3.

For proteome profiling, whole hippocampal lysates were analyzed by label-free quantitative liquid chromatography–mass spectrometry using data independent acquisition (DIA). A total of 6,116 proteins were identified. Differential protein expression analysis between miR-132 KD and control hippocampi (Figure 2A and Table S2) revealed broad but subtle effects on protein levels, which did not retain statistical significance after correction for multiple testing (Benjamini-Hochberg), similarly to previous reports on miRNA-dependent regulatory effects.^73^^,^^74^ Given that miRNAs are negative regulators of their targets, we selected the top 5% upregulated proteins (corresponding to 306 proteins). Next, we intersected this set with the previously extracted list of 384 *in silico* predicted miR-132 targets (Figure 2B). On this basis, we identified 10 putative miR-132 targets of interest, which include proteins expressed in both neurons and glia or only in glial populations (Table S1).

We next performed scRNAseq analysis in miR-132 KD and control hippocampi by employing a droplet microfluidics platform (10X Genomics Chromium) (Table S3). Cells were sampled from whole hippocampus, while additional excitatory neurons and astrocytes were obtained by FACS from Thy1-YFP transgenic mice. After quality control and filtering, we recovered data from 19,705 high-quality single cell transcriptomes. We observed high abundance of oligodendrocytes, microglia and astrocytes in our dataset, whereas only small numbers of distinct neuronal subtypes were retrieved (Figures 2C–2E), in-line with previous studies reporting the difficulty to isolate intact neuronal cells from the adult brain.^75^^,^^76^ All acquired datasets were integrated using Harmony,^77^ and the resulting integrated 14 clusters were annotated via established marker genes on uniform manifold approximation and projection (UMAP) plots (Figures 2C, 2D, and S2C).

We performed differential gene expression analysis in miR-132 KD and control cells, using a two-sided Wilcoxon rank-sum test with Bonferroni correction for multiple testing. For each of the main identified cell types, we calculated the amount of significantly differentially expressed genes (DEGs) (Wilcoxon rank-sum test, Bonferroni-corrected p value <0.05) (Figure 2E and Table S3), and extracted potential miR-132 targets among the identified upregulated DEGs. Significant transcriptional changes and miR-132 targets were found in microglia and oligodendrocytes (microglia, 78 DEGs and 1 predicted target; oligodendrocytes, 160 DEGs and 3 predicted targets), while we were not able to depict potential miR-132 targets in the neuronal and astrocytic populations, in accordance with the limited numbers of DEGs (astrocytes, 2 DEGs; granule cells, 1 DEG) (Figure 2E).

We then asked whether miR-132 knockdown exerts consistent effects on individual proteins and mRNAs in mouse hippocampus. To this end, we performed Pearson correlation analysis to compare directionality of the changes in protein and pseudo-bulked (upon *in silico* pooling of all cells) gene expression by plotting the logarithmic fold change (logFC) of gene/protein pairs shared between proteomics and transcriptomics (Figure S2A). Expression profiles of individual proteins/mRNAs did not show concordant changes.

We finally investigated whether the knockdown of miR-132 in the hippocampus may induce systematic effects on gene expression classified in specific biological pathways, despite the weak effects on individual protein and mRNA targets. We performed pathway enrichment analysis among the most differentially expressed (both upregulated and downregulated) proteins and genes using DAVID (Figures 2F and S2B). GO biological process analysis (Fisher’s Exact test, FDR-adjusted p value <0.05) confirmed again a representation of processes linked to general miRNA functioning, such as translation and mRNA processing, both in the proteomic and the cell type-specific transcriptomic datasets. Furthermore, functional terms previously associated with miR-132 were significantly enriched, including processes related to cell cycle (oligodendrocyte, OL; granule cell, GC; pyramidal neuron, PyN), neuron projection development (oligodendrocyte, OL; astrocyte, AST; granule cell, GC), nervous system development (oligodendrocyte, OL; astrocyte, AST; granule cell, GC), neuron differentiation (astrocyte, AST), and cytoskeleton organization (granule cell, GC; pyramidal neuron, PyN) (Figure 2F).^23^^,^^28^^,^^35^^,^^49^^,^^78^^,^^79^ Interestingly, we also identified biological processes that had not been previously reported in association with miR-132, involving endocytosis (oligodendrocyte, OL; astrocyte, AST; granule cell, GC; pyramidal neuron, PyN), apoptosis (oligodendrocyte, OL; microglia, MG; astrocyte, AST), and lipid metabolic process (Proteomics; astrocyte, AST). KEGG pathway enrichment analysis showed significant enrichment in endocytosis- (Proteomics; oligodendrocyte, OL; microglia, MG; granule cell, GC; pyramidal neuron, PyN) and autophagy- (oligodendrocyte, OL; microglia, MG; granule cell, GC; pyramidal neuron, PyN) related processes, and a strong representation of pathways linked to neurodegeneration (Transcriptomics) and synaptic signaling (Proteomics; oligodendrocyte, OL; astrocyte, AST; granule cell, GC; pyramidal neuron, PyN), formerly associated with miR-132^20^^,^^28^^,^^31^^,^^40^^,^^80^ (Figure S2B).

### miR-132 overexpression alters gene expression programs in both neuronal and immune-related pathways

**Figure 3:**
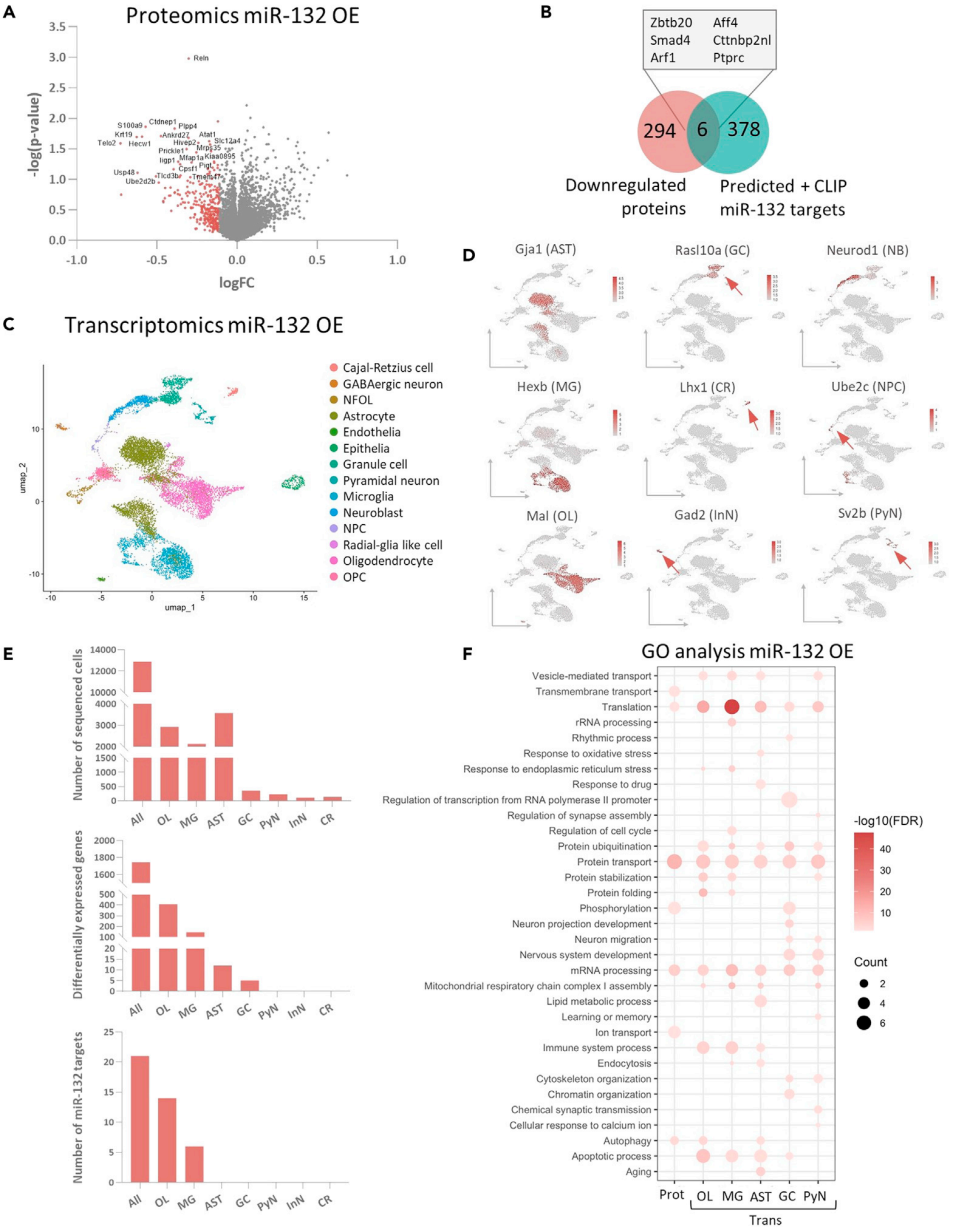
Impact of miR-132 overexpression in hippocampal cells (A) Differentially expressed proteins upon miR-132 OE presented in a volcano plot. The top 5% of proteins anticorrelated to miR-132 are indicated in red. (B) Identification of putative miR-132 targets from intersection with 5% most downregulated proteins and the list of identified predicted miR-132 targets. (C) UMAP visualizing 12,893 isolated mouse hippocampal miR-132 OE and corresponding control single-cell transcriptomes. Cells are colored by identified cell type. NFOL, newly formed oligodendrocyte; NPC, neuronal precursor cell; OPC, oligodendrocyte precursor cell. (D) UMAP plots colored by the normalized expression levels of cell type-specific marker genes used for cluster annotation. Arrows indicate the cell cluster of interest. (E) Number of cells, number of significant DEGs (Wilcoxon rank-sum test using Bonferroni for p value correction, adjusted p value <0.05 considered significant), or amount of putative miR-132 targets identified by intersection with predicted miR-132 targets, as included in the scRNAseq analysis. Counts correspond to all cells pseudo-bulked together (All) or to each cell type. (F) GO biological processes significantly enriched in differentially expressed proteins (Prot) or genes (Trans) in distinct cell types. Count represents the % of included proteins/genes that are part of each process. Color represents significance, top 20 GO terms are displayed (Fisher’s Exact test, p values corrected with FDR, adjusted p value <0.05 considered significant). AST, astrocyte; GC, granule cell; NB, neuroblast; MG, microglia; CR, Cajal-Retzius neuron; NPC, neuronal precursor cell; OL, oligodendrocyte; InN, inhibitory/GABAergic neuron; PyN, hippocampal pyramidal neuron. See also Figures S1, S2, Tables S2, and S3.

miR-132 overexpression could potentially elicit stronger effects in proteomic and transcriptomic profiles and is arguably more relevant in a therapeutic context.^81^ Thus, wild-type adult mice were ICV-injected with mimic (miR-132 mimic, miR-132 OE) oligonucleotides to overexpress miR-132. Control animals were infused with corresponding control (Control) oligonucleotides (Figures S1C and S1I). We confirmed that average miR-132 levels increased in all main cell types (Figure S1F), and hippocampi were subjected to bulk proteomics and scRNAseq, as before. For proteomics analysis, a total of 5,995 proteins were identified. Differential protein expression analysis comparing miR-132 OE and control did not result in significantly changing proteins after correction for multiple testing (Benjamini-Hochberg) (Figure 3A and Table S2). We then selected the top 5% downregulated proteins (corresponding to 300 proteins) and identified 6 putative miR-132 targets by overlaying with the list of *in silico* predicted targets (Figure 3B). Again, the identified proteins showed expression in both neuronal and glial populations in a previously published scRNAseq dataset in mouse hippocampus^72^ (Table S1).

We then characterized the transcriptional response using scRNAseq, obtaining a total of 12,893 single-cell transcriptomes after initial quality assessment and filtering (Figures 3C, 3D, and S2C). Most of the significant transcriptional changes and the only putative miR-132 targets were again identified in oligodendrocytes and microglia (Wilcoxon rank-sum test, Bonferroni-corrected p value <0.05) (oligodendrocytes, 408 DEGs and 14 predicted targets; microglia, 147 DEGs and 6 predicted targets; astrocytes, 12 DEGs, 0 predicted targets; granule cells, 5 DEGs, 0 predicted targets) (Figure 3E and Table S3). Similarly to the miR-132 KD datasets, there was no correlation between changes in individual gene/protein pairs (Figure S2A). GO biological processes and KEGG pathways significantly enriched after miR-132 OE were previously associated with miR-132 [e.g. cell cycle (microglia, MG), learning and memory (pyramidal neuron, PyN), cytoskeleton organization (granule cell, GC; pyramidal neuron, PyN) and neuronal projection development (granule cell, GC)], but also, similarly to miR-132 KD, linked to apoptosis (oligodendrocyte, OL; microglia, MG; astrocyte, AST; granule cell, GC) and the immune response [immune system process (oligodendrocyte, OL; microglia, MG; astrocyte, AST), antigen processing and presentation (oligodendrocyte, OL)] (Fisher’s Exact test, FDR-adjusted p value <0.05) (Figure 3F and S2B).

### Characterization of putative miR-132 targets in the hippocampus

**Figure 4:**
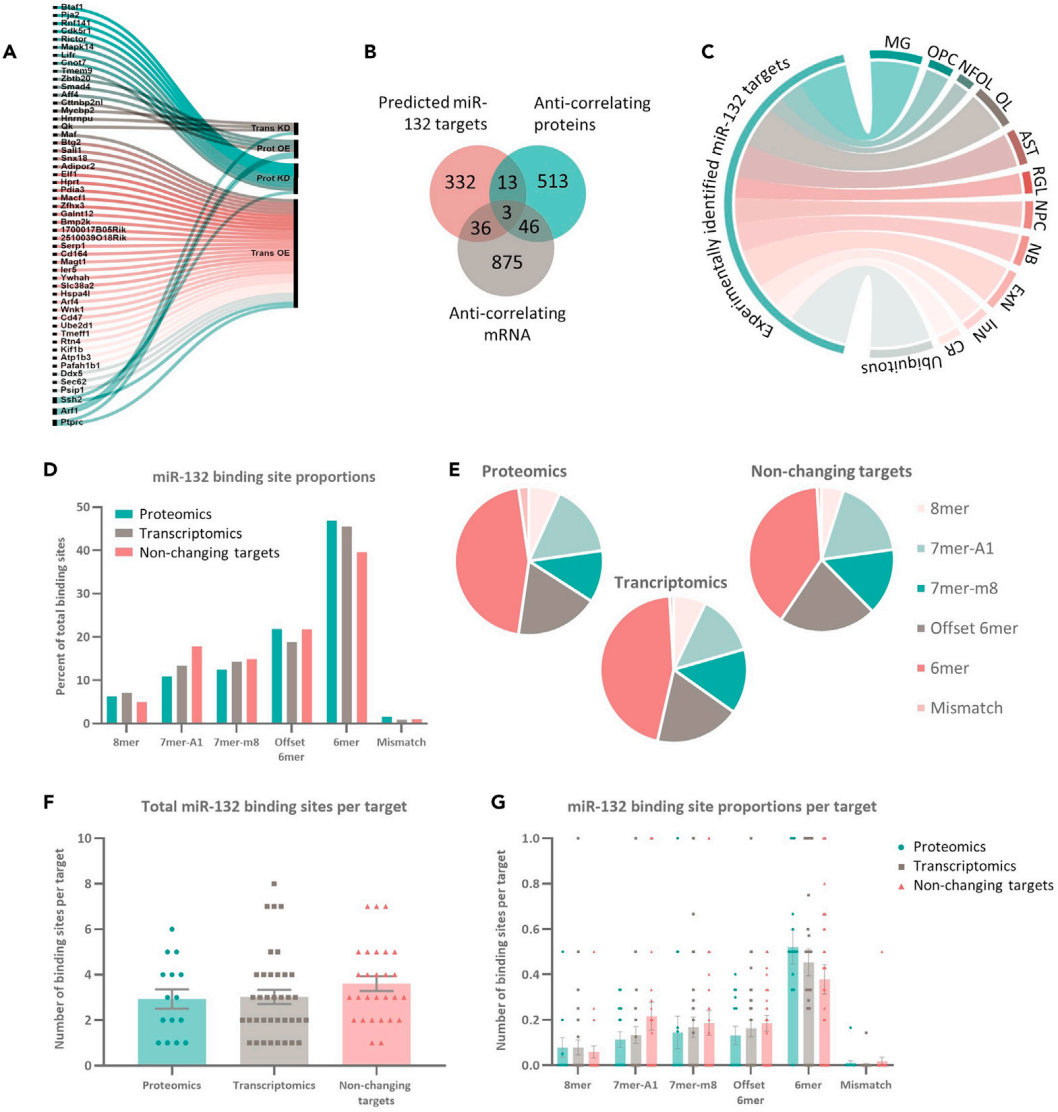
Characterization of putative miR-132 targets and miR-132 binding sites (A) List of 52 experimentally identified miR-132 targets linked to the dataset they were derived from. Trans, Transcriptomics; Prot, Proteomics. (B) Identification of putative miR-132 targets from proteomic and transcriptomic datasets by intersection with the list of *in silico* predicted miR-132 targets (Figure 1A). (C) Circos plot showing cell type-specific expression of 52 identified putative miR-132 targets. (D and E) Percentage of different types of miR-132 binding sites present in miR-132 targets that are derived from proteomic (Proteomics) or transcriptomic (Transcriptomics) datasets, or that are predicted miR-132 targets but not changing in any of the datasets (Non-changing targets). (F) Each data point represents the total amount of miR-132 binding sites present per target. (G) Proportions of different types of binding sites present per target. Each data point represents the proportion of the indicated binding site type per target. Proteomics, N = 15; Transcriptomics, N = 37; Non-changing targets, N = 28. Values are presented as mean ± SEM. See also Table S1.

Next, we took a closer look into the predicted miR-132 targets identified in each of the datasets. Even though a total of 52 putative miR-132 targets were identified in all the datasets combined, only three predicted targets (Ptprc, Arf1, and Ssh2) were common between proteomics (miR-132 KD/OE combined) and transcriptomics (miR-132 KD/OE combined) (Figures 4A, 4B, and Table S1). When considering the list of the 52 putative miR-132 targets, we observed an increase of experimentally identified putative targets expressed in microglia compared to the full set of 384 *in silico* predicted targets (12.9% in the experimental dataset compared to 4.8% in the *in silico* dataset) (Figure 4C). For example, *Ptprc*, which is also one of the three common miR-132 targets between proteomics and transcriptomics, is a myeloid cell marker, showing specific microglial expression in the hippocampus.^72^^,^^82^^,^^83^^,^^84^ Together, these findings suggest that a subset of the identified putative miR-132 targets possibly drives gene expression programs in microglial populations.

In an attempt to understand the lack of correlation between the miR-132 targets identified in the proteomic and the transcriptomic datasets, we explored in more detail the type and prevalence of miR-132 binding sites in the 3′UTR of the putative targets.^1^ First, we considered the proportions of binding site types present in targets that changed in the protein or RNA analysis and in predicted miR-132 targets that did not change in any of the datasets (non-changing targets) (Figures 4D, 4E, and Table S1). Apart from a trend for an increase of 6-mer seeds in the experimentally identified targets compared to the non-changing subset, no striking differences in the proportion of binding site types across datasets were observed. We did, however, notice an overall lower abundance of perfect 8-mer seed sequences, and higher abundance of 6-mer seeds across datasets, confirming previous observations on miRNA seed type proportions in an *in vitro* large-scale RNA-seq dataset.^60^ We then compared the total number of binding sites per target (Figures 4F, 4G, and Table S1). Again, the total amount of binding sites present per target did not differ across datasets, suggesting that seed (type) abundance cannot explain the differential effects of miR-132 on individual targets in our proteomics and transcriptomics datasets.

### miR-132 regulates microglial activation *in vivo*

**Figure 5:**
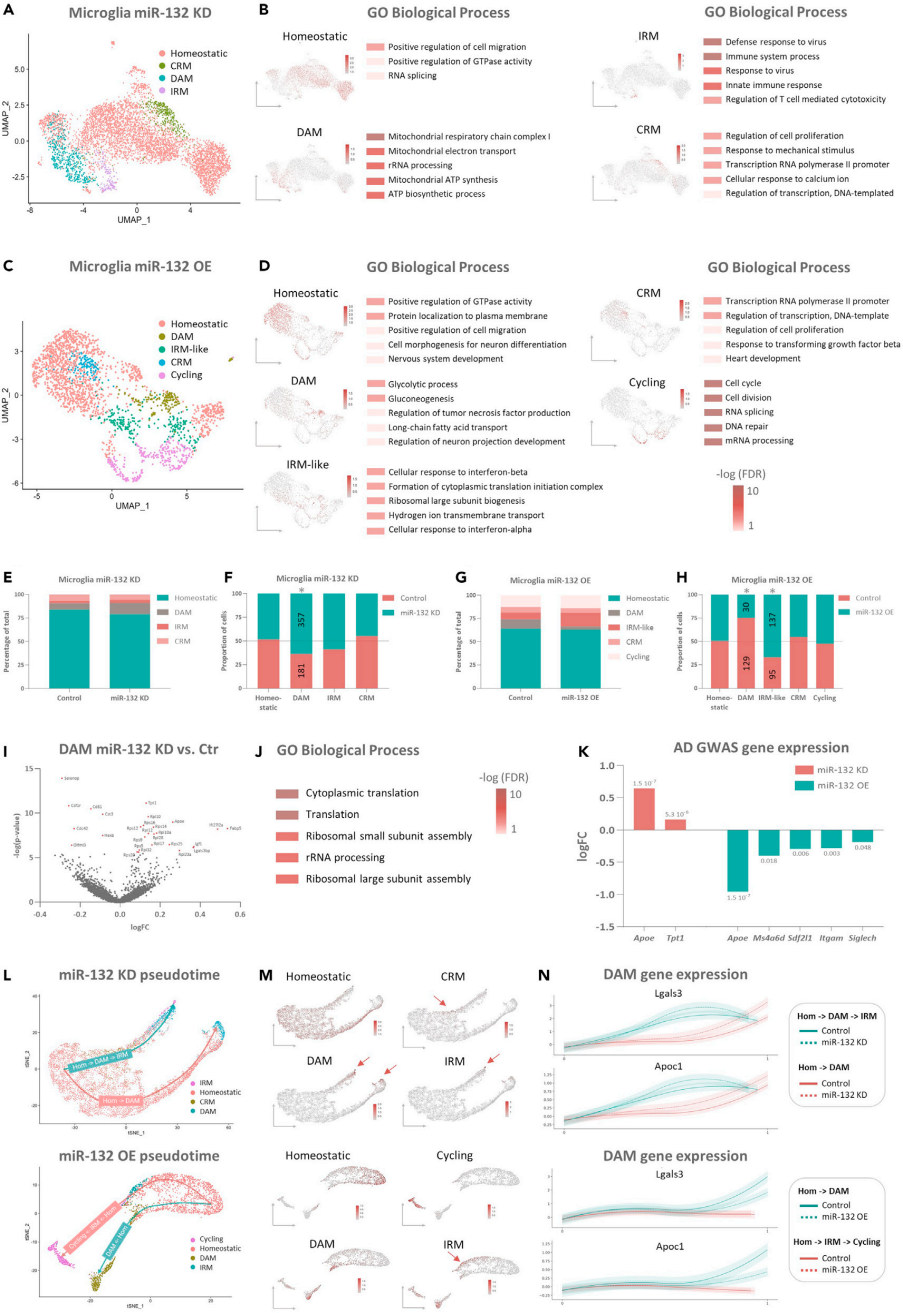
miR-132 drives microglial cell state transitions (A and C) Subset microglial population from miR-132 KD (A) or miR-132 OE (C) and corresponding control cells visualized on UMAP, colored according to identified clusters. (B and D) UMAP plots colored based on the signature score of the combined gene set that characterizes each individual microglial subpopulation in miR-132 KD (B) or miR-132 OE (D). Top 5 unique GO biological processes of significantly enriched genes are provided per subtype; color is indicated according to significance (Fisher’s Exact test, p values corrected with FDR, adjusted p value <0.05 considered significant). (E and G) Normalized percentage of cells present in distinct cellular states per condition in miR-132 KD (E) or miR-132 OE (G). (F and H) Normalized percentage of cells present in distinct cellular states per cellular state in miR-132 KD (F) or miR-132 OE (H). Significance indicated with an asterisk (Chi-squared test, miR-132 KD DAM: p value = 0.0062; miR-132 OE DAM: p value = 0.0000004; miR-132 OE IRM: p value = 0.0007). The number of cells present in miR-132 KD/OE or control condition is depicted on the graph. (I) Volcano plot showing DEGs comparing miR-132 KD cells to their corresponding controls in the DAM state. Significant DEGs indicated in red (Wilcoxon rank-sum test using Bonferroni for p value correction, adjusted p value <0.05 considered significant). (J) GO biological processes significantly enriched in the top 500 most DEGs, with color indicating significance (Fisher’s Exact test, p values corrected with FDR, adjusted p value <0.05 considered significant). (K) AD GWAS gene (adapted from^153^), p value cutoff set at 0.01) (*Apoe*, *Tpt1*, *Ms4a6d*, *Sdf2l1*, *Itgam*, *Siglech*) expression comparing miR-132 KD or OE cells with corresponding controls in the microglial population. Adjusted p values indicated (Wilcoxon rank-sum test using Bonferroni for p value correction, adjusted p value <0.05 considered significant). (L) Single-cell trajectories of microglial cells obtained by pseudotime ordering using Palantir. Color according to identified clusters. (M) UMAP plots colored based on the signature score of combined gene sets defining microglial cellular states. Arrows indicate populations of interest. (N) Gene expression of the DAM-associated genes *Lgals3* and *Apoc1* along pseudotime. Color represents the pseudotime branch and type of line the experimental condition. Homeostatic/Hom, homeostatic microglia; DAM, disease-associated microglia; IRM, interferon-response microglia; CRM, cytokine-response microglia; Cycling, cycling microglia. See also Figure S3 and Table S4.

To characterize the microglial population present in our dataset, we subclustered all microglial cells and annotated them based on published datasets^85^^,^^86^^,^^87^^,^^88^ (Table S4, “ModuleScore”). We identified five distinct microglial subsets, annotated as homeostatic microglia, disease-associated microglia (DAM), interferon-response microglia (IRM), cytokine-response microglia (CRM) and cycling microglia (Figures 5A–5D, S3A, S3D, S3E, S3H, and Table S4). Even though we did identify a microglial subpopulation expressing IRM-related genes (*Ifitm3*, *Ifi27L2a,* and *Isg15*) in the miR-132 OE dataset, this cluster did not recapitulate the full IRM response (and was therefore indicated as “IRM-like”). Next, we interrogated the GO biological processes associated with the significantly enriched DEGs in each subcluster (Figures 5B, 5D, and Table S4). Consistent with their role in immune surveillance, homeostatic microglia-enriched genes were related to cell migration. DAM-related genes were associated with mitochondrial energy production and glucose metabolism, which is in line with their highly metabolically active phenotype and the metabolic shift from oxidative phosphorylation to glycolysis upon activation.^89^^,^^90^^,^^91^^,^^92^^,^^93^ Both IRM and IRM-like populations were enriched for immune response-related pathways, while as expected, cycling microglia showed a strong enrichment for genes involved in cell cycle and division. CRM cells upregulated genes related to cell proliferation and transcription; at a closer look, we found them to also highly express a gene signature recently associated with *ex vivo* activated microglia^94^ (exAM; Figures S3B, S3F, and Table S4), suggesting that they may represent a microglial subpopulation triggered by the cellular dissociation process involved in the scRNAseq workflow. Integration of the microglial datasets from the miR-132 KD and OE experiments (Figure S3L) confirmed that cells from different samples and experiments are largely distributed across microglial subclusters, except for the cycling microglial population, which was only identified in the miR-132 OE experiment.

As miR-132 overexpression and knockdown triggered significant (Wilcoxon rank-sum test, Bonferroni-corrected p value <0.05) transcriptional responses in microglia (Table S4 for subcluster-specific expression changes), we next assessed whether these signatures could induce a phenotypic switch across microglial cell states. We therefore performed cell state composition analysis to determine the amount of cells present in each cell state, comparing miR-132 OE or miR-132 KD to their respective controls (Figures 5E–5H). Interestingly, the DAM cluster was expanded upon miR-132 knockdown (scProportion test, Chi-squared test: p value = 0.0062) (Figure S3C), whereas, reversely, it decreased upon miR-132 overexpression (scProportion test, Chi-squared test: p value = 0.0000004) (Figure S3G). In addition, increased miR-132 levels resulted in significantly more cells in the IRM-like subcluster (scProportion Test, Chi-squared test: p value = 0.0007) (Figure S3G), suggesting a potential miR-132-regulated phenotypic shift from DAM to an IRM-like state. Next, we analyzed the DEGs (Wilcoxon rank-sum test, Bonferroni-corrected p value <0.05) and corresponding GO biological processes (Fisher’s Exact test, FDR-adjusted p value <0.05) comparing miR-132 OE or KD to their respective controls, focusing on the DAM subcluster, which has high relevance for AD^85^^,^^88^^,^^95^ (Figures 5I, 5J, S3I, S3J, and Table S4). Knockdown of miR-132 triggered significant upregulation of DAM-associated genes (e.g. *Apoe*, *Fabp5,* and *Lgals3bp*), and downregulation of homeostatic microglial genes (e.g. *Csf1r*, *Hexb,* and *Cst3*), consistent with the observed increase in the population of DAM cells. Interestingly, we did not detect any significant DEGs after miR-132 overexpression in the DAM subpopulation, putatively due to the small total number of DAM cells in the miR-132 OE dataset (miR-132 OE, 30 DAM cells; Control, 129 DAM cells) (Figures 5H, S3I, and S3J). miR-132-responsive transcripts in DAM showed enrichment in ribosomal genes associated with translation, in-line with previous reports profiling the DAM phenotype.^87^^,^^96^ *Apoe*, one of the main risk factors of late-onset AD and a top expressor of the DAM transcriptional signature, was (even though not a predicted miR-132 target) among the top 4 most significantly upregulated genes upon miR-132 KD and, reversely, among the top 8 most downregulated genes after miR-132 OE in the microglial population, further supporting a role for miR-132 in gene expression programs regulating microglial cell state transitions (Figure 5K).^85^^,^^88^^,^^97^^,^^98^^,^^99^ In addition, other AD genome-wide association studies (GWAS) genes were significantly differentially expressed in our microglial population upon miR-132 KD (*Tpt1*, p value = 5.3 10^–6^) or miR-132 OE (*Ms4a6d*, p value = 0.018; *Sdf2l1*, p value = 0.006; *Itgam*, p value = 0.003; *Siglech*, p value = 0.048), suggesting an indirect regulation of AD risk genes by miR-132 (Figure 5K). Even though only significant in the miR-132 KD experiment, *Trem2*, another critical risk factor for AD and key modulator of microglial function,^100^^,^^101^^,^^102^ showed a trend for anticorrelation to miR-132 levels upon both overexpression and knockdown of miR-132 (Table S3). Of note, miR-132 depletion significantly induced inflammatory gene expression signatures also in IRM (*Ptgs1*, *Hsp90b1*) and homeostatic (*Lgals3bp*) microglial populations, while the expression of *Ctsl*, a DAM-associated gene, was significantly upregulated in homeostatic microglia. Reversely, miR-132 supplementation led to a significant reduction in inflammation-related genes in IRM-like (*Hsp90ab1*, *Hsp90aa1*, *Hspa8*) and homeostatic microglia (*Cmtm8*, *Nav3*, *Tmem173*) (Table S4).

To further understand the inhibitory effect of miR-132 on the DAM response, we performed single-cell trajectory analysis using Palantir.^103^ Pseudotime ordering of the microglial population revealed the existence of two distinct phenotypic routes from homeostatic toward cellular states corresponding to DAM, IRM, and cycling microglia (Figures 5L, 5M, and S3K). We then compared the expression profile of two DAM-associated genes (*Apoc1* and *Lgals3*) along pseudotime between miR-132 KD or miR-132 OE cells and their corresponding controls (Figure 5N). As expected, DAM gene expression collectively increased along the transcriptional route toward DAM. Interestingly, DAM gene expression levels were higher in miR-132 KD and lower in miR-132 OE cells compared to their controls, suggesting that, besides regulating the maintenance of the final pool of cells present in the DAM transcriptional profile, miR-132 also regulates DAM-related transcriptional programs during microglial transitions between homeostatic and DAM activation states. Employing the same approach as described for microglial cells, we also observed a decrease in the proportion of reactive oligodendrocytes upon miR-132 OE, an effect which was, however, not confirmed when miR-132 was knocked down (Figures S4, S5, and Table S5).

### miR-132 shifts human iPSC-derived microglia from a disease-associated toward a homeostatic state

**Figure 6:**
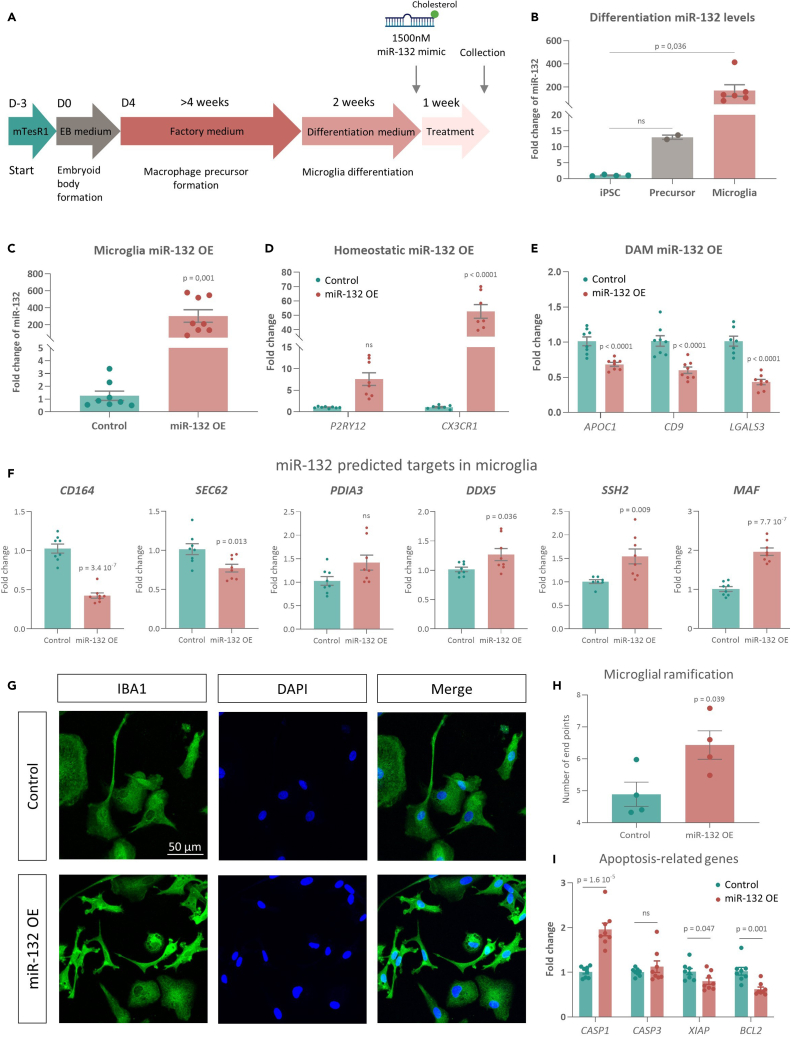
Human iPSC-derived microglia acquire a homeostatic phenotype upon miR-132 supplementation (A) Schematic representation of the differentiation protocol of human iPSCs into microglia. Cells were treated with cholesterol-conjugated miR-132 mimic or corresponding control oligonucleotide (1500 nM) for one week. (B) Semi-quantitative real-time PCR of miR-132 levels along microglial differentiation. N = 2–6 biological replicates. (C) Semi-quantitative real-time PCR of miR-132 levels in differentiated microglia after one week of treatment with a miR-132 mimic. N = 8 biological replicates. (D and E) Semi-quantitative real-time PCR of marker genes characteristic of homeostatic microglia (*P2RY12* and *CX3CR1*) (D) or DAM (*APOC1*, *CD9* and *LGALS3*) (E) N = 7–8 biological replicates. (F) Semi-quantitative real-time PCR of predicted miR-132 targets identified in the scRNAseq miR-132 OE experiment, as either downregulated in the microglial population (*SSH2*, *PDIA3*, *DDX5*, *SEC62*) or in the pseudo-bulked cells and also highly expressed in microglia (*MAF* and *CD164*).^72^ N = 8 biological replicates. (G) Immunolabeling of IBA1 (green) and DAPI (blue) in miR-132- or control-treated iPSC-derived microglia. Merge indicates images obtained from overlaying images separately acquired with distinct channels. (H) Morphological analysis of miR-132- or control-treated human iPSC-derived microglia, as quantified by the number of endpoints per cell. Each data point represents the average number of endpoints of 50 quantified cells, derived from two independent iPSC-to-microglia differentiations. (I) Semi-quantitative real-time PCR of apoptosis-related genes comparing miR-132-treated human iPSC-derived microglia to controls. N = 8 biological replicates. Values are presented as mean ± SEM. In B, one-way ANOVA with Bonferroni correction was applied. In C, F and H, Student’s *t* test was used. In D, E and I two-way ANOVA with Tukey’s correction was used. See also Figure S1.

We next validated our results in human iPSC-derived microglial cultures (Figure 6A). miR-132 levels increased along microglial differentiation (∼13-fold increase comparing macrophage precursors to iPSCs, ∼170-fold increase comparing microglia to iPSCs) (Figure 6B), supporting a functional role for miR-132 in microglial maturation. Interestingly, the increase in miR-132 levels was similar to that observed in human iPSC-derived neurons, further pointing toward important endogenous functions of miR-132 in microglia (Figure S1K). Notably, microglial cells in culture form a heterogeneous population, recapitulating several distinct transcriptional phenotypes linked to disease, and adapting in general a more activated phenotype.^86^^,^^104^^,^^105^ To investigate the potential effects of miR-132 on cellular state transitions in human iPSC-derived microglia, we treated differentiated microglia with a cholesterol-conjugated miR-132 mimic (or a control oligonucleotide) to increase miR-132 levels (Figures 6A and 6C). No changes in miR-132 levels upon mock-treatment and in miR-212 levels upon miR-132 mimic treatment were observed (Figures S1L–S1M). To profile microglial states, we monitored the expression levels of markers of homeostatic microglia (*P2RY12* and *CX3CR1*) and DAM (*APOC1*, *CD9,* and *LGALS3*)^85^^,^^86^^,^^106^ (Figures 6D and 6E). Increase of miR-132 levels significantly upregulated homeostatic microglial markers and downregulated DAM-associated genes, consistent with the miR-132-dependent anti-DAM response we observed in mouse hippocampus. Next, to gain insights into molecular targets that may be directly regulated by miR-132 in human microglia, we monitored the expression levels of several predicted miR-132 targets that we previously identified in the scRNAseq analysis (Figure 4). Specifically, we focused on the predicted targets that were downregulated either in the microglial population (*SSH2*, *PDIA3*, *DDX5*, *SEC62*) or in the pseudo-bulked cells and were additionally highly expressed in microglia (*MAF* and *CD164*) (Figure 6F).^72^ *SEC62* and *CD164* were significantly downregulated upon miR-132 increase, suggesting their putative conservation as direct miR-132 targets in human iPSC-derived microglia. Interestingly, *DDX5*, *SSH2,* and *MAF*, which have been previously linked to microglial homeostasis,^107^^,^^108^^,^^109^ were all upregulated upon miR-132 overexpression (Figure 6F), suggesting that they may be indirectly targeted by miR-132 to promote a homeostatic microglial phenotype. In addition, we assessed microglial morphological changes upon miR-132 increase, through immunolabeling of the microglial marker IBA1 (Figure 6G). Control oligonucleotide-treated cells displayed a round, amoeboid morphology, typical of activated or reactive microglia *in vitro*. In contrast, microglia treated with the miR-132 mimic exhibited pronounced ramified morphology, as quantified by a significant increase in cellular endpoints (Figure 6H), characteristic of homeostatic microglia partaking in immune surveillance through extension and retraction of mobile processes.^110^^,^^111^ Together, these findings confirm that increase of miR-132 levels drives the transition of human microglia from a DAM-like state to a more homeostatic phenotype. Lastly, to gain further insights into miR-132-regulated pathways in iPSC-derived microglia, we monitored apoptosis-related genes, as the apoptotic biological process was one of the gene ontologies significantly enriched in microglia both in the miR-132 OE and KD scRNAseq experiments in mouse hippocampus (Figures 2F and 3F). Caspase 1 (*CASP1*), a pro-apoptotic marker gene, was significantly upregulated upon miR-132 OE, while two anti-apoptotic regulators, *BCL2* and *XIAP*, were downregulated compared to control-treated cells (Figure 6I). Together, these data indicate that miR-132 may impact microglial phenotypes via-among others-the regulation of apoptotic programs both in mouse brain and in human microglial cultures.

## Discussion

Here, we employed a combinatorial proteomics and transcriptomics approach to profile broad regulatory effects of miR-132 in mouse hippocampus. Bulk proteomics analysis upon miR-132 overexpression and knockdown did not identify any significantly changing proteins. Also, transcriptional alterations in astrocytic and neuronal populations were limited. Yet, miR-132 modulation induced significant transcriptional responses in microglia and oligodendrocytes.

Currently, miRNA function is mostly inferred from studies using a “one miRNA-one target” approach. Yet, cumulative evidence from genome-wide studies suggests that miRNAs mainly function as regulators and fine-tuners of molecular networks.^50^^,^^112^^,^^113^ In addition, miRNA-mediated repression of single targets is often relatively small (20–30%) and largely depends on the cellular context.^56^^,^^57^^,^^114^^,^^115^ However, the profiling of broad miRNA-regulated gene networks (involved in cellular stress, apoptosis, inflammation, synaptic plasticity, and neuronal network activity) in mammalian brain has recently provided proof-of-concept for the utility of network-based approaches to drug discovery in neurological diseases.^50^^,^^116^

We observed limited effects on individual putative miR-132 targets upon miR-132 modulation in the mouse hippocampus. In addition, we did not depict significant correlation between individual transcripts and proteins across experimental conditions. Several factors could account for these findings, including -among others-differential (cell type-specific) effects of miR-132 on protein and mRNA targets, low overall correlation between proteome and transcriptome,^117^ technical differences in sensitivity, resolution and noise between the bulk proteomics, and scRNAseq approaches. Concordant changes in the mRNA and protein levels of miRNA targets are mostly observed for targets repressed by more than 50% (log2 > 0.58) by a miRNA, upon nearly perfect miRNA/mRNA binding^41^ and primarily in *in vitro* systems. Notably, in both our proteomics and transcriptomics datasets, we observed an overall lower frequency of perfect 8-mer seed sequences in putative miR-132 targets, which could be indicative of potentially smaller repression rates, and thus, potentially explain the lack of consistent effects between proteome and transcriptome. In addition, the total amount or type of binding sites present per target did not differ across our datasets. These observations suggest that perhaps other factors (possibly related to the cellular microenvironment, the 3′UTR context and accessibility or the existence of non-canonical binding sites and alternatively spliced mRNA isoforms, and biological noise) may determine target prioritization for miR-132 targeting in the complex hippocampal tissue.^1^^,^^118^^,^^119^^,^^120^^,^^121^^,^^122^ In iPSC-derived microglia, two predicted miR-132 targets identified by scRNAseq in mouse hippocampus were significantly downregulated upon induction of miR-132 levels. SEC62 has been implicated in protein translocation in the endoplasmatic reticulum,^123^ while CD164 positively affects chemotaxis.^124^ Yet, how these changes contribute to the observed phenotypic shift still remains to be determined.

miR-132 has been previously proposed to act as a NeurimmiR, a collective term referring to miRNAs acting at the interface between the neuronal and the immune system.^125^ With respect to its neuronal functions, miR-132 has been implicated in neuronal morphogenesis and survival, synaptic plasticity, and cognition.^23^^,^^28^^,^^31^^,^^33^^,^^34^^,^^35^^,^^80^ We recently reported a pro-neurogenic role for miR-132 in the adult hippocampus, where it regulates progenitor proliferation, neuronal differentiation, and maturation of newly born neurons, while also providing neurotrophic and neuroprotective support.^35^ On the other hand, miR-132 overexpression suppresses the inflammatory response in monocytes, macrophages, and astrocytes *in vitro*, while it also represses the secretion of pro-inflammatory cytokines by targeting acetylcholinesterase in the periphery.^126^^,^^127^^,^^128^ Interestingly, microglial miR-132 levels increase in human and rat brain after epileptic seizures, suggesting a possible role for miR-132 in the neuroinflammatory response under these conditions.^129^ Yet, there is currently no evidence of direct regulation of the innate immune response by miR-132 in the brain. Cell type-specific expression profiling showed that miR-132 is highly expressed in hippocampal microglia already at baseline. Earlier evidence also suggested that miR-132 is among the top 14% most abundant miRNAs in microglia.^130^ Taken that only the most highly expressed miRNAs in a given cell type have been proposed to significantly regulate targets in that particular cellular context,^131^ miR-132 expression levels in microglia suggest that it may have the potential to effectively repress its targets and thereby exert a functional regulatory effect on microglial function. Indeed, miR-132 overexpression restricted the proportion of a disease-associated microglial population, while, reversely, miR-132 depletion significantly increased this microglial fraction. miR-132 levels were reversely associated with DAM gene expression signatures along unbiased pseudotime ordering of microglia from a homeostatic to a DAM cell state. Validation of these observations in human iPSC-derived microglia (where endogenous miR-132 levels increase approximately 200-fold compared to iPSCs), confirmed that miR-132 shifts human microglia from a disease-associated toward a homeostatic phenotype. Interestingly, an anticorrelation between miR-132 levels and inflammatory gene signatures was not only observed in DAMs, but also in other microglial subclusters, like homeostatic microglia, *in vivo*. We also observed significant changes in the levels of apoptosis-related genes in the iPSC-derived microglial cultures, suggesting that miR-132 could mediate its effects on microglial activation and homeostasis via -among others-regulating apoptosis in microglial (sub)populations.^132^^,^^133^^,^^134^^,^^135^ Whether the impact of miR-132 regulation on microglial populations *in vivo* is partially also effectuated by non-cell-autonomous signaling from other cell types onto microglia, like for instance via neuronal exosomes,^136^ cannot be ruled out. miR-132 overexpression experiments in neurons co-cultured with microglia could provide valuable insights in that regard. In addition, we observed a similar effect of miR-132 in oligodendrocytes, where we identified a reactive population characterized by high *Serpina3n* and *C4b* expression.^137^^,^^138^ miR-132 overexpression resulted in a decrease of this reactive subcluster and repressed inflammatory genes within this population. Together, these findings support a role for miR-132 in the immune response in the hippocampus.

Recently, comprehensive single-cell transcriptomic studies in AD mouse models and human brain revealed complex activation profiles of distinct microglial cell states with significant roles in AD pathogenesis.^85^^,^^86^^,^^87^^,^^88^^,^^139^^,^^140^ Low proportions of activated microglial subpopulations are also present in wild-type mouse brain, as we have observed, suggesting that these clusters represent physiological states of microglia.^88^ DAMs are a microglial subset expressing many of the AD risk genes identified in genome-wide association studies (GWAS), like *TREM2* and *APOE*, the largest risk factor for AD.^85^^,^^88^^,^^97^^,^^101^^,^^102^^,^^141^^,^^142^^,^^143^ Interestingly, we observed a significant reverse association between the levels of miR-132 and those of *ApoE* and other AD risk genes in the whole microglial population, suggestive of the modulating effect of miR-132 on the DAM phenotypic response.

Our goal here was to understand which targets and biological processes miR-132 would affect in the hippocampus, as a first step toward exploring what would be the limitations of targeting miR-132 in AD. We and others have previously shown that miR-132 exerts a wide range of neuroprotective functions, ameliorates amyloid, and Tau pathologies and improves memory in AD mouse models.^23^^,^^32^^,^^33^^,^^35^ While the current work mainly confirms that miR-132 supplementation can exert widespread effects across several cellular populations in the brain, it also suggests that such responses are likely going to be subtle. One possible avenue of concern is the regulatory effect of miR-132 overexpression on the equilibrium between different microglial cell states. Microglias are important carriers of genetic risk in AD^144^ and it is, therefore, currently unclear whether fixing microglia in a more homeostatic phenotype would be beneficial or detrimental in the disease.^145^^,^^146^^,^^147^^,^^148^ Targeting miR-132 overexpression or knockdown specifically to microglial (sub)populations in the brain may therefore be of particular biological and therapeutic relevance in the future.

### Limitations of the study

Our study has three main limitations. First, given the relative subtle changes, limited sample size and inter-individual variability could have partially confounded some of our observations. Proteomic changes upon miR-132 knockdown or overexpression did not retain significance after correction for multiple testing. For instance, inflammation-related proteins, like Lgals3bp, Ifit1/3, and Serpina3n, even though not significant, were among the top 5% anticorrelated to miR-132. While this may reflect the overall more subtle effect of miRNAs on proteome^43^^,^^149^ than on the transcriptome, it could also be the result of the dilution of cell type-specific effects in whole hippocampal lysates (which contain different brain structures, like blood vessels and ventricles, and several other cell types, such as -among others- endothelial and epithelial cells, pericytes, macrophages), which might be overcome by using larger sample sizes. Most importantly, we did not observe many significant transcriptional changes in any of the neuronal populations analyzed via scRNAseq upon miR-132 OE (despite the robust miR-132 overexpression), which is in contrast with the well documented neuronal functions of miR-132, and could therefore most likely be attributed to the technical difficulties of isolating distinct neuronal subpopulations. This could have contributed to the relatively small numbers of neurons in our scRNAseq dataset, which may have compromised the power of the downstream analyses. In addition, during the process of neuronal isolation, axons and dendrites, which represent cellular compartments with high miR-132 expression,^36^^,^^150^^,^^151^ may be lost, impacting the precise estimation of miR-132 (and putatively also target) levels both at baseline, but also after miR-132 manipulation. Moreover, the observed lack of miR-132 knockdown in neurons could potentially reflect a less productive oligonucleotide uptake, leading to inefficient target knockdown.^152^ Some of the enriched biological pathways that were identified in the dataset and were related to neuronal function do not necessarily reflect cell-autonomous signaling, as other cell types like microglia or oligodendrocytes might affect neuronal function. Interestingly, only small numbers of significant DEGs and no putative miR-132 targets were identified in the astrocytic cluster, despite processing adequate numbers of isolated cells (miR-132 KD, 3456; miR-132 OE, 3575) [i.e. comparable numbers to oligodendrocytes (miR-132 KD, 6004; miR-132 OE, 2928) and microglia (miR-132 KD, 5603; miR-132 OE, 2127)]. These observations may suggest that astrocytes are potentially less amenable to miR-132 regulation, at least under these experimental conditions.

Second, we have here employed wild-type mice and healthy donor iPSC-derived microglial cultures, in order to assess the regulatory output of miR-132 under physiological conditions. While essential for defining a therapeutic window in the future, our findings warrant further research into the immunomodulatory effects of miR-132 in an AD-relevant context.

Third, our proteomics and transcriptomics approaches do not enable discrimination between direct and indirect miR-132 effects. Binding assays (e.g., reporter assays, CLIP) can identify direct targets, yet do not offer any information on the functional output of these interactions. On the other hand, genome-wide expression profiling of the transcriptome or the proteome upon miRNA modulation cannot discriminate between primary and secondary effects but does allow for global understanding of the biological pathways regulated by a miRNA.^42^ Taking these into consideration, we here employed an integrative, multimodal approach to broadly profile the functional implications of miR-132 regulation in the hippocampus. While such a distinction between direct and indirect miR-132 regulatory effects would be further informative from a mechanistic perspective, our validation experiments in human iPSC-derived microglia confirmed that a shift from DAM to homeostatic microglia is one of the main functional effects of miR-132 regulation in the hippocampus.

## STAR★Methods

### Key resources table

**Table:** 

REAGENT or RESOURCE	SOURCE	IDENTIFIER
**Antibodies**		

Mouse anti-ACSA-2-APC	Miltenyi Biotec	Cat#130-116-142; RRID: AB_2727422
Rat anti-CD11b-BV421	BD	Cat#562605; RRID: AB_11152949
Mouse anti-O4-PE	Miltenyi Biotec	Cat#130-117-357; RRID: AB_2733886
Rabbit anti-IBA1	WAKO	Cat#019–19741; RRID:AB_839504

**Chemicals, peptides, and recombinant proteins**		

Actinomycin D	Sigma-Aldrich	Cat#A1410-5 MG
Lipofectamine 2000	Thermo Fisher Scientific	Cat#11668019

**Critical commercial assays**		

miRVana Paris Kit	Life Technologies	Cat#AM1556
miRNeasy Micro Kit	Qiagen	Cat#217084
miRCURY LNA RT Kit	Qiagen	Cat#339340
Superscript II reverse transcriptase	Thermo Fisher Scientific	Cat#18064071
Neuron Isolation Kit, mouse	Miltenyi Biotec	Cat#130-115-390
Adult Brain Dissociation Kit, mouse and rat	Miltenyi Biotec	Cat#130-107-677
ChromiumSingleCell3 v3	10X Genomics	Version 3, CG000183
ChromiumNextGEMSingleCell3	10X Genomics	Version 3.1, CG000204

**Deposited data**		

Single-cell RNA sequencing (FASTQ and metadata)	Gene Expression Omnibus, NCBI	GEO: GSE230333

**Experimental models: Cell lines**		

Human induced pluripotent cell line (iPSC)	Sigma-Aldrich	Cat#IPSC0028

**Experimental models: Organisms/strains**		

C57BL/6 (C57BL/6JRj)	The Jackson Laboratory	RRID:IMSR_JAX:000664
Thy1-YFP-16 mice (B6.Cg-Tg(Thy1-YFP)16Jrs/J)	The Jackson Laboratory	RRID:IMSR_JAX:003709

**Oligonucleotides**		

miR-132 antagomiR (locked nucleic acid (LNA)-, 3′-cholesterol-modified oligonucleotide)	Qiagen	Cat# 500150; Design ID: 280412
Scramble LNA antagomiR control	Qiagen	Cat# 500150; Design ID: 280407
miR-132 miRIDIAN mimic; UAAC AGUCUACAGCCAUGGUCG (MIMAT0000426)	Dharmacon	Cat# C-300599-06
Control miRIDIAN mimic; UCACA ACCUCCUAGAAAGAGUAGA (MIMAT0000039)	Dharmacon	Cat# CN-001000-01
Primer mmu-miR-132-3p; UAA CAGUCUACAGCCAUGGUCG	Qiagen	Cat# 204129
Primer mmu-miR-212-3p; UAAC AGUCUCCAGUCACGGCCA	Qiagen	Cat# 206022
mRNA primers (for full sequence, see Table S6)	IDT	Custom primer sets

**Software and algorithms**		

FIJI	Schindelin et al.^166^	https://fiji.sc/
Encori	Li et al.^62^	https://starbase.sysu.edu.cn/
Magma	De Leeuw et al.^165^	https://ctg.cncr.nl/software/magma
Palantir	Setty et al.^103^	https://github.com/dpeerlab/Palantir
David	Huang et al.^64^	https://david.ncifcrf.gov/
Seurat	Butler et al.^163^	Version 4.1.1, https://satijalab.org/seurat/
Scanpy	Wolf et al.^159^	Version 1.8.1 https://scanpy.readthedocs.io/en/stable/
Spectronaut	Biognosys	Spectronaut 14

### Resource availability

#### Lead contact

Further information and requests for resources and reagents should be directed to and will be fulfilled by the lead contact, Evgenia Salta (e.salta@nin.knaw.nl).

#### Materials availability

This study did not generate new unique reagents.

### Experimental model details

#### Animals

All animal experiments were approved by the ethical committees of UZ Leuven and KU Leuven. Mice were bred in-line with the institutional guidelines and housed under standard 12-h light-dark cycles. Food and water were provided *ad libitum*. Adult wild type C57Bl/6 or Thy1-YFP mice (B6.Cg-Tg(Thy1-YFP)16Jrs/J, RRID:IMSR_JAX:003709) of 7–9 weeks old were used. For the proteomics study, only male mice were used, while for the transcriptomics both male and female mice were approximately equally distributed across experimental groups. Littermates were randomly assigned to experimental groups.

#### Microglial differentiation of human induced pluripotent stem cells (iPSCs)

Human induced pluripotent stem cells (iPSCs) (Sigma Ctrl iPSCs P5 24/7/21, Cat# IPSC0028, female, identified through short tandem repeats) were differentiated into microglia at 37°C as previously described.^154^ Briefly, iPSCs were thawed in a matrigel-coated (Corning Matrigel hESC-Qualified, Cat#354277) 6-well plate in mTeSR1 (Cat#85850, Stemcell) supplemented for the first 24 h with 10 μM Y-27632 (Rock inhibitor, Cat#72304, Stemcell). When confluent, iPSCs were transferred to an AggreWell 800 (Cat#34815, Stemcell) for Embryoid Body (EB) formation at 4 × 10^6^ cells per well. Cells were fed daily and kept in mTeSR1 supplemented with 50 ng/mL BMP4 (Cat#PHC9531, Thermo Fisher Scientific), 50 ng/mL VEGF (Cat#100–20, Peprotech), and 20 ng/mL SCF (Cat#130-096-693, Miltenyi Biotec). Next, four-day EBs were differentiated in 6-well plates (approximately 15 EBs/well) in X-VIVO15 (Cat#BE02-060F, Lonza), supplemented with 2 mM Glutamax (Cat#35050038, Life Technologies), 50 μM β-mercaptoethanol (Cat#31350010, Thermo Fisher Scientific), 50 U/ml penicillin/streptomycin (Cat#P4333-100 mL, Sigma-Aldrich), 25 ng/mL Il-3 (Cat#PHC0035, Thermo Fisher Scientific), and 100 ng/mL M-CSF (Cat#PHC9501, Thermo Fisher Scientific), with fresh medium added weekly. Macrophage precursors were harvested after at least 1 month and passed through a 40 μm cell strainer, spun down at 400 g for 5 min and resuspended in differentiation medium for differentiation into microglia. Differentiation medium consisted of Advanced DMEM/F12 (Cat#12634–010, Thermo Fisher Scientific) supplemented with 1 mM Glutamax, 50 μM β-mercaptoethanol, 50 U/ml penicillin/streptomycin, 100 ng/mL IL34 (Cat#200–34, Peprotech) and 10 ng/mL GM-CSF (Cat#PHC2013, Thermo Fisher Scientific). Microglia differentiation medium was changed approximately 3 times per week.

#### Neuronal differentiation of human induced pluripotent stem cells (iPSCs)

Human induced pluripotent stem cells (iPSCs) (Sigma Ctrl iPSCs P5 24/7/21, Cat# IPSC0028, female, identified through short tandem repeats) were differentiated into neurons at 37°C according to a dual SMAD protocol as previously described.^155^ Briefly, once iPSCs reached 70% confluence, cells were enzymatically dissociated with accutase (Cat#A6964, Sigma-Aldrich) and single cells were replated (3 × 106 cells/well) in Matrigel-coated (Corning Matrigel hESC-Qualified, Cat#354277) 6-well plates in mTeSR1 (Cat#85850, StemCell), supplemented with 10 μM Y-27632 (Rock inhibitor, Cat#72304, StemCell) for 24 h. Once cells reached 100% confluence, neural induction was initiated on day *in vitro* (DIV) 0 by replacing mTeSR1 (Cat#85850, StemCell) with neural maintenance medium, containing 50% neurobasal medium (Cat#21103–049, Thermo Fisher Scientific), 50% DMEM:F12 GlutaMAX (Cat#31331–028, Thermo Fisher Scientific), 2% B27 supplement (Cat#17504–044, Thermo Fisher Scientific), 1% N2 (Cat#17502–048, Thermo Fisher Scientific), 1% Glutamax (Cat#35050061, Thermo Fisher Scientific), 1% sodium pyruvate (Cat#11360070, Thermo Fisher Scientific), 1% MEM non-essential amino acid solution (NEAA, Cat#11140–035, Thermo Fisher Scientific), 1% penicillin/streptomycin (Cat#4333, Sigma-Aldrich), 50 μM 2-mercaptoethanol (Cat#31350010, Thermo Fisher Scientific) and 0.025% insulin (Cat#I19278, Sigma-Aldrich), supplemented with 10 μM SB431542 (SMAD inhibitor, Cat#S4317, Sigma-Aldrich) and 1 μM LDN-193189 (Noggin analog, Cat#130-106-540, Miltenyi Biotec). Cells were maintained in neural induction medium until DIV10 with daily medium change. From DIV10 to DIV24, cells were kept in neural maintenance medium and neuroepithelium was passaged mechanically every 4 to 5 days to enrich for neural rosettes. At approximately DIV24, neural rosettes were dissociated into single cells using accutase (Cat#A6964, Sigma-Aldrich) and single cells were replated (5 × 106 cells/well) in laminin-coated (Cat#L2020, Sigma-Aldrich) 6-well plates. At approximately DIV28, cells were frozen as neural progenitor cells (NPCs). To continue maturation, NPCs were thawed in neural maintenance medium supplemented with 10 μM Y-27632 (Rock inhibitor, Cat#72304, Stemcell) for 24 h in laminin-coated (Cat#L2020, Sigma-Aldrich) plates. Once 100% confluent, cells were replated using accutase (Cat#A6964, Sigma-Aldrich) in PLO- (Polyornithine, Cat#P4957, Sigma-Aldrich) and laminin (Cat#L2020, Sigma-Aldrich) coated plates and kept in neural maintenance medium supplemented with 20 ng/mL brain-derived neurotrophic factor (Cat#78005, StemCell), 20 ng/mL glial cell line-derived neurotrophic factor (Cat#78058, StemCell), 200 μM cAMP (Cat#73882, StemCell) and 200 μM ascorbic acid (Cat#A5960, Sigma-Aldrich). Cells were incubated at 37°C under 5% CO2 with 50% of medium changed twice a week. At DIV65, neurons were collected for RNA extraction.

### Method details

#### Intracerebroventricular (ICV) injections

Mice were anesthetized using isoflurane and fixed in a stereotactic frame. A cannula was implanted unilaterally in the right hemisphere at position of injection (AP-0.1 mm, ML-1.0 mm, and DV-3.0 mm) and mice were left to recover for approximately 1 week. For miR-132 knockdown (miR-132 KD), mice were injected with 3 μL (0.33 nmol/μL) of miR-132 antagomiR (locked nucleic acid (LNA)-, 3′-cholesterol-modified oligonucleotide) (Exiqon, QIAGEN) resuspended in artificial cerebrospinal fluid (aCSF) (Harvard Apparatus). Control mice received a scrambled LNA oligonucleotide in aCSF. For miR-132 overexpression (miR-132 OE), mice received 150 pmol of either a miR-132 mimic or a negative control oligonucleotide (Dharmacon, Horizon Discovery) in a 3 μL mix with lipofectamine 2000 (at a 1:1 vol ratio) (Cat#11668019, Thermo Fisher Scientific). Due to the differences in chemical modifications of the mimic and antagomiR oligonucleotides, different treatment times were employed for each experiment.^156^^,^^157^^,^^158^ One week (miR-132 KD) or 48 h (miR-132 OE) after injection, mice were sacrificed and the injected right hippocampus was microdissected. For proteomics analysis, the hippocampi were snap frozen in liquid nitrogen and stored at −80°C until further processing. For transcriptomic analysis, mice were first perfused and isolated hippocampi were immediately further processed (see ‘hippocampal cell dissociation’).

#### Proteomics

##### Preparation of samples for mass spectrometry

For miR-132 overexpression, 25 male wild type mice were injected with negative control oligonucleotide (n = 12) or miR-132 mimics (n = 13). For miR-132 knockdown, two independent cohorts of mice were employed, yielding a total of 38 mice: control (n = 19) and miR-132 KD (n = 19). Hippocampal samples were dissected, snap frozen and stored at −80°C. Cell lysates were prepared by addition of 3 mL urea lysis buffer (8 M Urea 20 nM HEPES, pH 8.0), and after mixing by pipetting, samples were subjected to 10 cycles of sonication in a 10°C water bath. Subsequently, cell lysates were cleared by centrifugation at 20000*g* at RT for 15 min and protein concentrations were measured by using Bradford assay. Samples were diluted by the addition of SDS to a final concentration of 5%, tris(2-carboxyethyl)phosphine (TCEP) to a final concentration of 10 mM and triethylammonium bicarbonate (TEAB) to a final concentration of 50 mM. Samples were alkylated in the dark for 1 h by the addition of 2-iodoacetamide (IAA) at 20 mM. Protein lysates were prepared for mass spectrometry using the s-trap mini plate format according to the manufacturer’s instructions (Cat#NC1828287, Protifi). In summary, for each sample 50 mg of protein was loaded onto an s-trap 96-well plate. Captured protein was washed 5 times with 200 mL of wash buffer (90% methanol with 100 mM TEAB, pH 7.1). Proteins were digested by the addition of 5 mg of trypsin to each sample in 50 mM ammonium bicarbonate. Samples were digested for 2 h at 47 °C. Once digestion was complete, peptides were eluted with 80 mL of 50 mM ammonium bicarbonate followed by 80 mL of 0.2% formic acid and lastly with the addition of 80 mL 50% acetonitrile with 0.2% formic acid. After the addition of each elution buffer, the plate was centrifuged at 1500 g for 2 min and the flowthrough was collected. Eluted peptides were dried by speedvac and suspended in 1% formic acid before quantification using the CBQCA assay (Cat#C6667, Invitrogen).

#### Mass spectrometry

For each sample, 1.5 mg of peptide was analyzed by DIA. Peptides were injected onto a nanoscale C18 reverse-phase chromatography system (UltiMate 3000 RSLC nano, Cat#ULTIM3000RSLCNANO, Thermo Fisher Scientific) and electrosprayed into an Orbitrap Exploris Mass Spectrometer (Thermo Fisher Scientific). For liquid chromatography, the following buffers were used: buffer A (0.1% (v/v) formic acid in Milli-Q water) and buffer B (80% (v/v) acetonitrile and 0.1% (v/v) formic acid in Milli-Q water). Samples were loaded at 10 μL/min onto a trap column (100 μm × 2 cm, PepMap nanoViper C18 column, 5 μm, 100 Å, Cat#17294434, Thermo Fisher Scientific) equilibrated in 0.1% trifluoroacetic acid (TFA). The trap column was washed for 3 min at the same flow rate with 0.1% TFA then switched in-line with a resolving C18 column (75 μm × 50 cm, PepMap RSLC C18 column, 2 μm, 100 Å, Cat#164563, Thermo Fisher Scientific). Peptides were eluted from the column at a constant flow rate of 300 nL/min with a linear gradient from 3% buffer B to 6% buffer B in 5 min, then from 6% buffer B to 35% buffer B in 115 min, and finally to 80% buffer B within 7 min. The column was then washed with 80% buffer B for 4 min and re-equilibrated in 3% buffer B for 15 min. Two blanks were run between each sample to reduce carry-over. The column was kept at a constant temperature of 50°C.

The data were acquired using an easy spray source operated in positive mode with spray voltage at 2.445 kV, and the ion transfer tube temperature at 250°C. The MS was operated in DIA mode. A scan cycle comprised a full MS scan (*m*/*z* range from 350 to 1650), with RF lens at 40%, AGC target set to custom, normalized AGC target at 300%, maximum injection time mode set to custom, maximum injection time at 20 ms, microscan set to 1 and source fragmentation disabled. MS survey scan was followed by MS/MS DIA scan events using the following parameters: multiplex ions set to false, collision energy mode set to stepped, collision energy type set to normalized, HCD collision energies set to 25.5, 27 and 30%, orbitrap resolution 30000, first mass 200, RF lens 40%, AGC target set to custom, normalized AGC target 3000%, microscan set to 1 and maximum injection time 55 ms. Data for both MS scan and MS/MS DIA scan events were acquired in profile mode.

#### Analysis of mass spectrometry data

Raw mass spec data files were searched employing Spectronaut (Biognosys) version 14.7.201007.47784 using the directDIA function. The following search settings were used: minimum peptide length 7, maximum peptide length 52, cleavage enzyme trypsin, maximum missed cleavages 2, protein and peptide FDR was set at 0.01, profiling and cross run normalization were disabled. Carbamidomethyl (C) was selected as a fixed modification while Acetyl (N-term), Deamidation (NQ) and Oxidation (M) were selected as variable modifications. Data were searched against a mouse database from Uniprot release 2020 06. This database consisted of all manually annotated mouse SwissProt entries along with mouse TrEMBL entries with protein level evidence and a manually annotated homologue within the human SwissProt database.

#### Hippocampal cell dissociation and fluorescence-activated cell sorting (FACS)

Following injection of miR-132 mimic/control oligonucleotides (miR-132 OE) or miR-132 antagomiR/control oligonucleotides (miR-132 KD), mice received an overdose of pentobarbital and were perfused with ice-cold aCSF. The aCSF was composed of (in mM): 93 NMDG, 2.5 KCl, 1.2 NaH2PO4, 30 NaHCO3, 20 HEPES, 25 glucose, 5 sodium ascorbate, 2 thiourea, 3 sodium pyruvate, 10 MgSO4, 0.5 CaCl2, and was adjusted to pH 7.4 and equilibrated in 95% O_2_ 5% CO_2_. After perfusion, right hippocampi were microdissected and placed in cold Hibernate A Low Fluorescence (BrainBits), supplemented with 5 μM Actinomycin D (Cat#A1410-5 MG, Sigma-Aldrich). Hippocampi were dissociated enzymatically using the Adult Brain Dissociation Kit, mouse and rat (Cat#130-107-677, Miltenyi Biotec) for 15 min at 37°C, with 5 μM Actinomycin D added. Subsequently mechanical dissociation was performed, cells were passed through a 70 μm cell strainer and washed with 10 mL ice-cold D-PBS supplemented with 0.5% BSA. Next, cells were centrifuged for 10 min at 300 g at 4°C and myelin and debris removal was performed according to manufacturer’s instructions. Afterward, cells were resuspended in D-PBS (0.5% BSA) and labeled with the eBioscience Fixable Viability Dye eFluor 780 (1:2000, Cat#65-0865-14, Thermo Fisher Scientific, irreversibly labels dead cells) and in run 2, 4, 5 also with Anti-mouse ACSA-2-APC (1:20, Cat#130-116-142, Miltenyi Biotec) and BV421 Rat Anti-CD11b (1:10, Cat#562605, BD). Cells were incubated for 30 min in the dark at 4°C, washed with 1 mL D-PBS (0.5% BSA) and spun down for 10 min at 300 g at 4°C. Subsequently, cells were suspended in 200 μL D-PBS (0.5% BSA) for FACsorting. Only alive cells were sorted, as dead cells were excluded based on their staining with the eFluor 780 dye. Neurons were sorted based on YFP expression. For astrocyte isolation, ACSA-2 positive microglia were excluded by selection based on both CD11b and ACSA-2 expression. According to the run, an Influx (BD) or MACSQuant Tyto (Miltenyi Biotec) were used for sorting and different cell types were isolated (Table S3).

Cells were kept on ice for the entire cell dissociation protocol except for 15 min at 37°C during enzymatic dissociation. To prevent any transcriptional changes due to the dissociation procedure *per se*,^94^ a transcription inhibitor (Actinomycin D) was added during tissue collection and cell dissociation steps, as indicated.

#### Isolation of main cell types from mouse hippocampus using magnetic activated cell sorting (MACS) and fluorescence-activated cell sorting (FACS)

Isolated hippocampi were initially processed as described in section ‘hippocampal cell dissociation’. After debris and myelin removal steps, cells were resuspended in 80 μL D-PBS supplemented with 0.5% BSA and FcR Blocking Reagent was added (1:10, Cat# 130-092-575, Miltenyi Biotec). After a wash step with 1 mL D-PBS (0.5% BSA), cells were stained with Non-Neuronal Cells Biotin-Antibody Cocktail (1:5), washed, and Anti-Biotin Micro-Beads (1:5) were added according to manufacturer’s instructions (Neuron Isolation Kit, mouse, Cat#130-115-390, Miltenyi Biotec). Labeled cells were loaded into a pre-rinsed LS column (Cat#130-042-401, Miltenyi Biotec) placed in a QuadroMACS Separator (Cat#130-090-976, Miltenyi Biotec). Flowthrough was collected and pooled with flowthough after washing the column with 2 × 1 mL D-PBS (0.5% BSA). This fraction contained unlabeled cells corresponding to the neuronal cell population. Columns were removed from the separator and flushed with 3 mL D-PBS (0.5% BSA), yielding a fraction containing the non-neuronal cell population.

The neuronal population was spun down at 5000 g for 5 min at 4°C and cell pellets stored at −80°C until further processing. The non-neuronal population was stained with eBioscience Fixable Viability Dye eFluor 780 (1:2000), anti-mouse ACSA-2-APC (1:20) for astrocyte isolation, BV421 Rat anti-CD11b (1:10) for microglial isolation, and anti-mouse O4-PE (1:50, Cat#130-117-357, Miltenyi Biotec) for oligodendrocyte isolation. Cells were incubated for 30 min in the dark at 4°C, spun down and resuspended in 200 μL D-PBS (0.5% BSA) for FACS. Astrocytes (ACSA-2+, CD11b-), microglia (CD11b+) and oligodendrocytes (O4+) were isolated on an Influx platform (BD), spun down and cell pellets stored at −80°C until further processing. Average percentages of sorted cells are depicted in Figure S1J.

#### Single-cell library preparation using the 10X chromium platform and sequencing

For scRNAseq, 10000–20000 cells were sorted per sample and further processing was conducted following manufacturer’s instructions (CG000183 ChromiumSingleCell3 v3 for RUN 1, 2, 3 and CG000204 ChromiumNextGEMSingleCell3 v3.1 for RUN 4, 5). Briefly, cells were loaded onto the Chromium Single Cell Chip B (RUN 1, 2, 3) or Chromium Next GEM Chip G (RUN 4, 5) for GEM formation and after consecutive steps of reverse transcription, cDNA clean-up and amplification, DNA libraries were generated. Libraries were loaded in an equimolar pool for sequencing on the HiSeq4000 or NovaSeq6000 platform (Illumina) aiming for 50000 reads/cell.

#### Data processing

##### Proteomics

Sample KD1-1 was determined as an outlier in an initial principal component analysis and was thus removed from count and metadata tables, and then proteins not observed in at least 75% of samples in each experimental group were removed from the count table. Counts were RPM normalized before running differential expression analysis via Limma-Voom between miR-132 OE/KD groups and corresponding control groups. Voom estimates the mean-variance relationship and computes observation-level weights before a linear model is fit for each protein (additionally, the 75% protein detection threshold was decided based on voom mean-variance distribution). Empirical bayes moderated t-tests were run to determine differential expression comparing miR-132 KD/OE to corresponding control-injected samples. Benjamini-Hochberg correction was applied to p values.

#### Single-cell RNA sequencing

Raw 10x Genomics data were processed using the Cellranger count command (v3.1.0), mapping to the GRCm38 assembly (ensembl 97 annotation). Filtered gene expression matrices were loaded into Scanpy (v.1.8.1)^159^ and further filtered to remove cells expressing less than 500 genes or with greater than 15% of UMIs being assigned to mitochondrial genes, Scrublet was then used to remove cells likely to be doublets (doublet_score >0.25), and finally, samples were then combined into two datasets, miR-132 OE and miR-132 KD. Following QC and filtering, samples were normalized to a total of 10000 UMIs per cell and log normalized; highly variable genes were detected using default parameters and the data were scaled to unit variance with a mean of 0, values greater than 10 were clipped. A principal component analysis was performed on the highly variable gene matrix and Harmony^77^ was used for batch correction between runs, 50 and 40 components were used respectively for miR-132 OE and miR-132 KD for downstream dimensionality reduction and clustering analyses. Clustering was performed at various resolutions using the Leiden algorithm and clusters were annotated with cell types based on their marker gene expression. Infiltrating immune cells segregated as distinct clusters and were removed from the analysis based on the expression of markers for monocytes (*Cd44*, *Fn1*, *Gda*), macrophages (*Atf3*, *Cfp*, *Serpinb8*), dendritic cells (*Flt3*) and lymphocytes (*Lck*).^84^^,^^160^^,^^161^ Cells from cell types we did not intend to sort per RUN, were excluded from further analysis [RUN 1: all hippocampal cells; RUN 2: only neurons and astrocytes; RUN 3: all hippocampal cells; RUN 4: only neurons and astrocytes; RUN 5: only neurons and astrocytes (Table S3)]. After removal of these cells, the analysis was repeated starting with the detection of highly variable genes, 46 and 42 principal components were used respectively for miR-132 OE and miR-132 KD for filtered datasets.

#### Identification of cell subtypes and differences in cell proportions across conditions

Processed and annotated datasets (miR-132 OE and miR-132 KD) were loaded into the Seurat pipeline^162^^,^^163^ using the SeuratDisk package (https://mojaveazure.github.io/seurat-disk/), for subclustering analysis. To better identify and annotate cell subtypes and states, the major cell types of interest (microglia and oligodendrocytes) were subset from the original dataset and processed separately using the subset function of Seurat. The Seurat package (v4.1.1) was used to perform linear dimensional reduction. 2000 highly variable genes were selected as input for PCA and significant PCs were identified based on the JackStrawPlot function. Strong first 10 (oligodendrocytes – miR-132 OE), 20 (neuronal cells – miR-132 OE/KD, microglia – miR-132 KD, oligodendrocytes – miR-132 KD), and 30 (microglia – miR-132 OE) PCs were used for UMAP to cluster the cells by FindClusters function with resolution 0.8–1.4. When multiple runs were performed (Table S3) data were integrated using Harmony^77^ to correct for run-specific batch effects. Clusters were identified by the expression of known cell type or cell state markers and by employing Seurat AddModuleScore function, using curated lists of features derived from previous publications (Tables S4 and S5).

Identification of DEGs among clusters and conditions was performed using the FindAllMarkers function (thresh.use = 0.25, test.use = “wilcox”) with the Seurat R package. We used the Wilcoxon rank-sum test (default), and genes with average expression difference >0.5 natural log with p value <0.05 were selected as marker genes. DEGs between miR-132 KD/OE versus control were identified using the same approach, with no threshold set. The significant DEGs were determined using the Wilcoxon rank-sum test with False Discovery Rate (FDR) correction using all genes in the dataset. Genes were considered significant at FDR <0.05.

To determine changes in cell subtype proportions of miR-132 KD/OE versus control, the number of cells per subtype was normalized against the total number of cells into the respective major cell type per condition, implemented by Cluster_Stats_All_Samples function of scCustomize package.^164^ Significant changes in proportions were determined using scProportionTest library (https://github.com/rpolicastro/scProportionTest) and Chi-squared test. Proportional changes were considered significant at p value <0.05.

#### Pseudotime

Palantir (v1.1)^103^ was used to perform pseudotime analysis. In short, microglial clusters along the lineage were extracted and an appropriate start cell was selected based on expression of markers expected early in the lineage, i.e. a microglial cell with the highest expression of homeostatic marker genes in this case. Palantir was then run on each subset using the Harmony corrected principal component analysis values as the base representation (n_components = 5, knn = 30, n_eigs = 3, num_waypoints = 500). After pseudotime calculation, Palantir objects were separated according to condition, in order to compare the effect of condition on pseudotime.

#### Pathway enrichment

To identify biological pathways regulated by miR-132 both at the level of the protein and RNA, we performed functional enrichment analysis using the Database for Annotation, Visualization and Integrated Discovery (DAVID).^64^^,^^65^ For proteomics and single-cell transcriptomics (divided in oligodendrocytes, microglia, astrocytes, granule cells and hippocampal pyramidal neurons), differentially expressed proteins or genes were ranked based on their p value and the top 1000 up- and downregulated proteins or genes were used as input for pathway analysis. For functional analysis of the identified 384 predicted miR-132 targets, all 384 targets were used as input. Both GO Biological Process and KEGG pathways were processed and the pathways with an FDR-adjusted p value <0.05 (Fisher’s Exact test, FDR-adjusted p values <0.05 considered significant) and a count of genes ≥1% were selected. Pathways were ranked based on their significance and the top 20 per condition (cell type for transcriptomics and proteomics) were selected for further processing. After pooling together pathways with high functional overlap, both gene count and significance of the largest subcategories were plotted.

To determine which biological pathways were specific to a certain cell state or type, we extracted all the marker genes per cell state/type that were upregulated and had an FDR-adjusted p value <0.05 (Fisher’s Exact test, FDR-adjusted p values <0.05 considered significant) and uploaded those into DAVID. GO Biological Processes were selected based on significance (FDR-corrected p value <0.05) and only the top 5 pathways that were unique to that cell state/type were displayed.

After differential analysis comparing miR-132 KD/OE versus control in DAM microglia and reactive oligodendrocytes, pathway analysis was performed as described here. Briefly, DEGs were ranked on p value and the top 500 anti-correlating (upregulated after miR-132 KD and downregulated after miR-132 OE) genes were used as input in DAVID. The top 5 GO Biological Processes that had an FDR-adjusted p value <0.05 (Fisher’s Exact test, FDR-adjusted p values <0.05 considered significant) were shown.

#### Identification and cell type-specific expression of miR-132 predicted targets

Identification of *in silico* predicted miR-132 targets was performed using Encori. Targets predicted by at least 2 out of 7 (microT, miRanda, miRmap, PITA, RNA22, PicTar, TargetScan) target prediction algorithms were intersected with experimental AGO-CLIP databases (using a high-confidence threshold, Table S1).

To determine the cell type-specific expression profile of miR-132 predicted targets, we used both our in-house mouse hippocampus scRNAseq dataset and the previously published mouse dentate gyrus dataset.^72^ Three independent scientists manually annotated the expression profile of the predicted targets based on relative expression distribution across cell types (http://linnarssonlab.org/dentate) and consensus expression was plotted (Table S1).

#### Microglial dataset integration

Microglia datasets from the two experiments (miR-132 OE and miR-132 KD) were integrated using the Seurat integration analysis (CCA algorithm) (Figure S3L). In order to do so, genes that were repeatedly variable across two datasets were selected with the function SelectIntegrationFeatures. Subsequently, cross-dataset cell-pairs that were in a matched biological state were identified using the function FindIntegrationAnchors followed by integrating these two datasets with the function IntegrateData. Dimension reduction analysis was performed on the integrated datasets with the function RunPCA and RunUMAP. The clustering analysis was accomplished with the functions FindNeighbors and FindClusters.

#### AD GWAS gene analysis

To identify possible miR-132-regulated AD risk loci, we made use of a recent study^153^ that reports a large genome-wide association meta-analysis of clinically diagnosed late-onset AD (94,437 individuals) to identify AD risk loci. MAGMA^165^ first maps SNPs to genes based on genomic proximity, then tests for association of the gene to a trait. Here, Stage 1 summary statistics generated by Kunkle et al.,^153^ were analyzed with MAGMA v1.07, using the NCBI GRCh37 build and the European 1000 genomes as references. We set the annotation window (genomic region) at 35 kb upstream and 10 kb downstream of the gene boundaries. We then converted the list of genes output from MAGMA to their mouse orthologs using Ensemble Biomart Release 94, selecting only genes with strict one-to-one orthologs. Employing an adjusted p value cutoff of 0.01, we extracted a list of 571 GWAS genes (Table S4, “AD GWAS genes”), and compared it to the significant DEGs identified in our microglial population in both miR-132 KD and miR-132 OE datasets.

#### miR-132 mimic treatment of human iPSC-derived microglia

Differentiated microglia were treated with a synthetic miR-132 mimic oligonucleotide (Batch ID 60374517, cholesterol-conjugated, Janssen Pharmaceutica) or corresponding control oligonucleotide at 1500 nM and collected after 1 week of treatment for RNA extraction or fixed on a coverslip with 4% PFA for immunocytochemistry.

#### RNA extraction, reverse transcription and semi-quantitative real-time PCR

RNA isolation from the mouse hippocampus was performed using the miRVana Paris Kit (Cat#AM1556, Life Technologies) according to the manufacturer’s instructions. Briefly, using 350 μL of cell disruption buffer (containing protease and phosphatase inhibitors) the tissue was homogenized. After denaturation, acid phenol:chloroform was added and the samples were incubated and spun down, resulting in the formation of 2 phases. The upper aqueous phase was supplemented with 1.25 volumes of 100% ethanol and loaded on miRVana spin columns. Following consecutive washing steps, RNA was eluted from the column and concentration and purity were measured using NanoDrop. For the isolated brain cells and collected iPSC-derived microglia, RNA was extracted using the miRNeasy Micro Kit (Cat#217084, Qiagen) following manufacturer’s instructions.

Reverse transcription of miRNAs was performed with 100 ng RNA using the miRCURY LNA RT Kit (Cat#339340, QIAGEN). For the reverse transcription of mRNAs, 200 ng RNA was processed using the Superscript II reverse transcriptase (Cat#18064071, Thermo Fisher Scientific).

For miRNAs, real-time semi-quantitative PCR was performed using the Sybr Green mastermix (Cat#1708880, QIAGEN) and miRCURY LNA primers (Cat#204129, QIAGEN). The mean expression of two small-RNA housekeeping genes, U6 snRNA (Cat#203907, QIAGEN) and RNU5G (Cat#203908, QIAGEN), was used for normalization. Coding transcripts were analyzed using the SensiFast Sybr No-Rox kit (Cat#BIO-98020, Bioline) and *GAPDH*, *UBC* and *PSMB4* were used as housekeeping genes for human mRNAs. *Gapdh* and *Actin* were used as housekeeping genes for mouse mRNAs. Primer sequences can be found in Table S6. Ct values were determined using the second derivative method and subsequently fold changes were calculated using the ΔΔCt method.

#### Immunocytochemistry of iPSC-derived microglia

PFA-fixed microglial cells on coverslips, were initially washed with 1% (v/v) Triton X-100 in PBS and incubated with blocking buffer, consisting of 1% (v/v) Triton X-100, 10% (v/v) normal goat serum in PBS, for 2 h at room temperature. The primary antibody (anti-IBA1 (Rabbit), 1:100, Cat#019–19741, WAKO) incubation was performed in 0.3% (v/v) Triton X-100, 3% (v/v) normal goat serum in PBS at 4°C overnight, followed by incubation with the secondary antibody (Goat anti-rabbit 488, 1:500, Cat#111-487-003, Dylight) for 2 h in the dark at room temperature. Finally, cells were washed with 1% (v/v) Triton X-100 in PBS, stained in the dark with DAPI (1:5000) and mounted in Mowiol.

#### Image acquisition, processing and analysis

Images of the miR-132-treated microglia were acquired using a Leica TCS SP8 X confocal microscope. Images were acquired at 20× magnification with an additional digital zoom of 2.66, speed of 100, and z stack size of 20 μm. For image processing, the FIJI software was employed.^166^ To obtain overlaid images from different channels, we used the ‘Merge Channel’ function in FIJI. The number of endpoints per cell was quantified from immunofluorescent confocal images of IBA1-immunolabelled treated iPSC-derived microglia using ImageJ.

### Quantification and statistical analysis

Statistical analysis was performed as indicated in the figure legends. Significance was set at (adjusted) p value threshold of 0.05.

## Data Availability

Single-cell RNA-seq data have been deposited at GEO and are publicly available as of the date of publication. Accession number is listed in the key resources table. Any additional information required to reanalyze the data reported in this paper is available from the lead contact upon request.

## Supplemental information

Document S1. Figures S1–S5 and Table S6

Table S1: Identification of miR-132 targets. Related to STAR Methods and Figures 1 and 4

Table S2: Proteins changing upon miR-132 overexpression or knockdown. Related to Figures 2 and 3

Table S3: Differentially expressed genes upon miR-132 overexpression or knockdown. Related to STAR Methods and Figures 2, 3 and 5

Table S4: Enriched and differentially expressed genes in microglial subpopulations upon miR-132 overexpression or knockdown. Related to STAR Methods and Figure 5

Table S5: Enriched and differentially expressed genes in oligodendrocytic subpopulations upon miR-132 overexpression or knockdown. Related to STAR Methods and Figures S4 and S5
