# Supplementary material for: microRNA-132 regulates gene expression programs involved in microglial homeostasis
Source: iScience. 2023 May 6;26(6):106829. doi: 10.1016/j.isci.2023.106829 (PMC10213004; doi:10.1016/j.isci.2023.106829)
Supplement: Document S1. Figures S1–S5 and Table S6 [file mmc1.pdf]

iScience, Volume *26*

Supplemental information

microRNA-132 regulates gene expression programs involved in microglial homeostasis

Hannah Walgrave, Amber Penning, Giorgia Tosoni, Sarah Snoeck, Kristofer Davie, Emma Davis, Leen Wolfs, Annerieke Sierksma, Mayte Mars, Taofeng Bu, Nicola Thrupp, Lujia Zhou, Diederik Moechars, Renzo Mancuso, Mark Fiers, Andrew J.M. Howden, Bart De Strooper, and Evgenia Salta

Supplemental figures

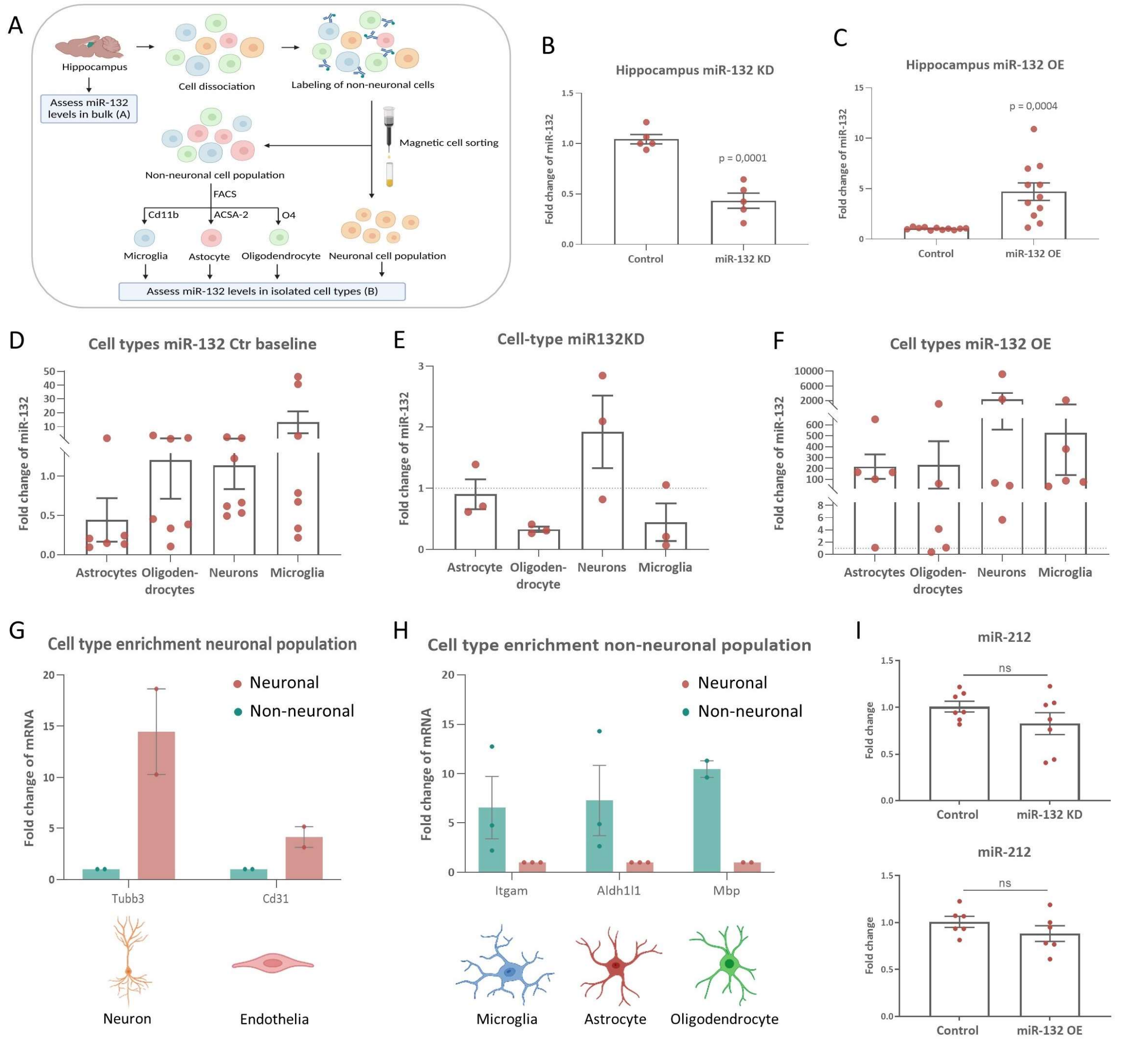

J

Cell type	% sorted
Neuron	37.94
Astrocyte	29.82
Microglia	19.06
Oligodendrocyte	13.18

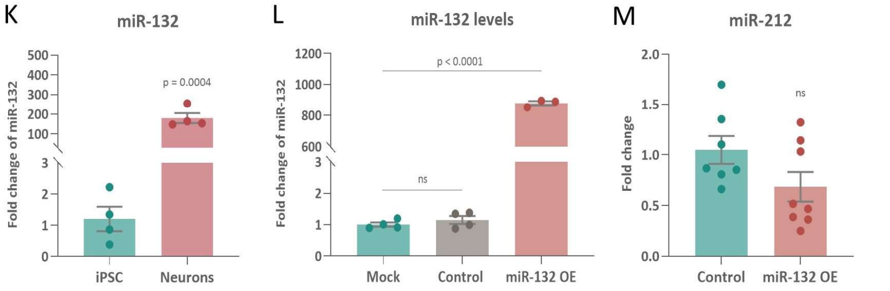

Figure S1. Hippocampal miR-132 expression profile. Related to STAR Methods and Figures 2, 3 and 6. A. Schematic representation of isolation protocol of four major brain cellular populations using MACS and FACS. **B.** Semi-quantitative real-time PCR of miR-132 levels in total hippocampus after intracerebroventricular injection with antagomiR (miR-132 KD) or control oligonucleotide. N = 5 mice per group. **C.** Semi-quantitative real-time PCR of miR-132 levels in total hippocampus after injection

with mimic (miR-132 OE) or control oligonucleotide. N = 11 mice per group. **D.** Semi-quantitative real-time PCR of miR-132 levels in isolated cell types of control-injected hippocampi. miR-132 levels in astrocytes, oligodendrocytes and microglia were normalized against neuronal levels. N = 6-7 independent experiments (each experiment contains 4-5 pooled hippocampi). **E.** Semi-quantitative real-time PCR of miR-132 levels in isolated cell types after injection with antagomiR (miR-132 KD) compared each time to corresponding control-injected samples. N = 3 independent experiments (each experiment contains 4-5 pooled hippocampi). **F.** Semi-quantitative real-time PCR of miR-132 levels in isolated cell types after injection with mimic (miR-132 OE) compared each time to corresponding control-injected samples. N = 5 independent experiments (each experiment contains 4-5 pooled hippocampi). Dotted lines in E, F represent control levels. **G, H.** Semi-quantitative real-time PCR of cell type-specific marker genes comparing neuronal and non-neuronal isolated fractions in mouse brain: *Tubb3* (neurons), *Cd31* (endothelia), *Itgam* (microglia), *Aldh1l1* (astrocytes) and *Mbp* (oligodendrocytes). N = 2-3. **I.** Semi-quantitative real-time PCR of miR-212 levels in total hippocampus after intracerebroventricular injection with antagomiR (miR-132 KD, top panel)/mimic (miR-132 OE, bottom panel) or control oligonucleotide. N = 6-7 mice per group. **J.** Average percentages (%) of neurons, astrocytes, microglia and oligodendrocytes sorted for miR-132 level quantification depicted in Figure S1D-F. N = 5 independent experiments. **K.** Semi-quantitative real-time PCR of miR-132 levels in human iPSCs and iPSC-derived neurons. N = 4 biological replicates. **L.** Semi-quantitative real-time PCR of miR-132 levels in iPSC-derived microglia after treatment with mimic (miR-132 OE), control oligonucleotide or medium (mock). N = 3-4 biological replicates. **M.** Semi-quantitative real-time PCR of miR-212 levels in iPSC-derived microglia after treatment with mimic (miR-132 OE) or control oligonucleotide. N = 7-8 biological replicates. Values are presented as mean ± SEM. In B, C, I, K and M Student's t-test was applied. In D-F, one-way ANOVA with Bonferroni correction was employed. In L, one-way ANOVA with Bonferroni correction was applied.

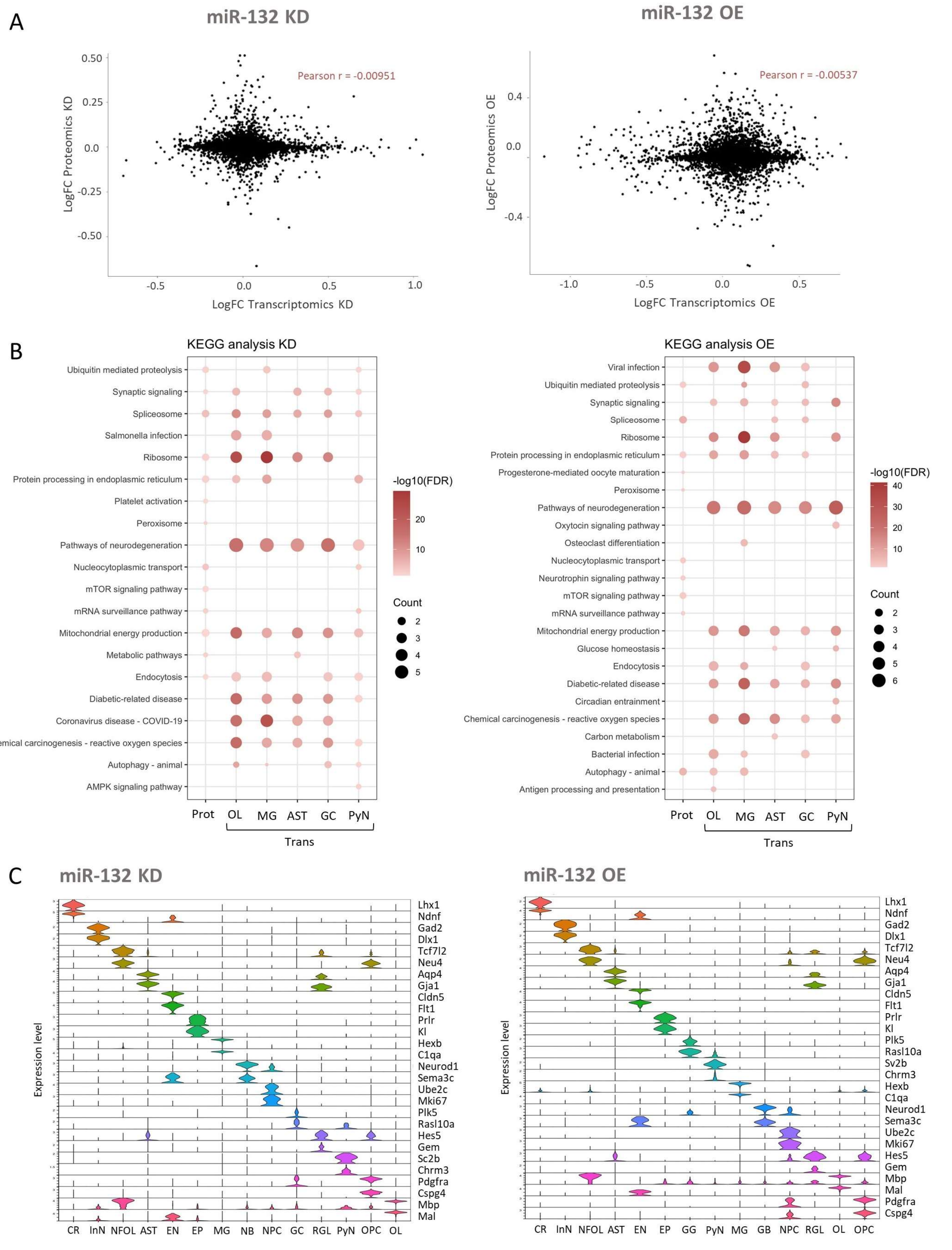

Figure S2. Correlation between proteomic and transcriptional miR-132 response. Related to Figures 2 and 3. A. Correlation analysis between the common protein and pseudo-bulked gene pairs. Logarithmic fold change (logFC) comparing miR-132 KD/OE with corresponding controls is plotted. Pearson correlation: R = -0.00951 (miR-132 KD) and -0.00537 (miR-132 OE). **B.** Significantly enriched KEGG pathways of differentially expressed proteins (Prot) or genes (Trans) in distinct cell types. Count

represents the % of included proteins/genes that are part of each process. Color represents significance and top 20 GO terms are displayed (Fisher's Exact test, p-values corrected with false discovery rate, adjusted p-value < 0.05 considered significant). **C.** Violin plots of normalized expression levels of cell type-specific marker genes in distinct cell clusters used for cluster annotation. OL, Oligodendrocyte; MG, microglia; AST, astrocyte; GC, granule cell; PyN, hippocampal pyramidal neuron; CR, Cajal-Retzius neuron; InN, inhibitory/GABAergic neuron; NFOL, newly-formed oligodendrocyte; EN, endothelial cell; EP, epithelial cell; NB, neuroblast; NPC, neuronal precursor cell; RGL, radial-glia like cell; OPC, oligodendrocyte precursor cell.

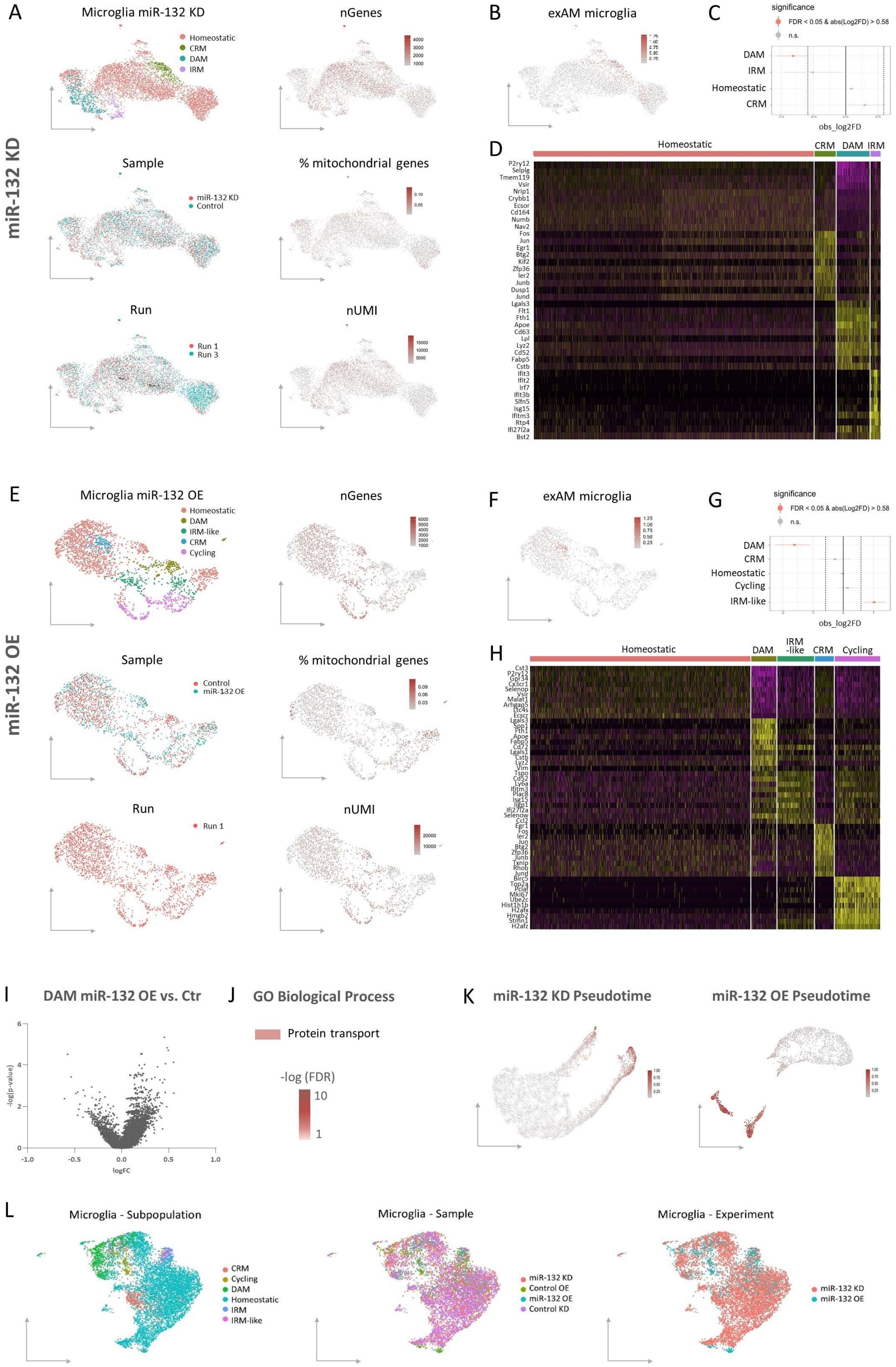
A
Microglia miR-132 KD
Homeostatic
CRM
DAM
IRM
nGenes
Sample
miR-132 KD
Control
% mitochondrial genes
Run
Run 1
Run 3
nUMI
miR-132 KD
B
exAM microglia
C
significance
FDR < 0.05 & abs(Log2FD) > 0.58
n.s.
DAM
IRM
Homeostatic
CRM
obs_log2FD
D
Homeostatic
CRM
DAM
IRM
P2ry12
Selplg
Tmem119
Vsir
Nrip1
Crybb1
Ecscr
Cd164
Numb
Nav2
Fos
Jun
Egr1
Btg2
Klf2
Zfp36
Ier2
Junb
Dusp1
Jund
Lgals3
Ftl1
Fth1
Apoe
Cd63
Lpl
Lyz2
Cd52
Fabp5
Cstb
Ifit3
Ifit2
Irf7
Ifit3b
Slfn5
Isg15
Ifitm3
Rtp4
Ifi27l2a
Bst2
E
Microglia miR-132 OE
Homeostatic
DAM
IRM-like
CRM
Cycling
nGenes
Sample
Control
miR-132 OE
% mitochondrial genes
Run
Run 1
nUMI
miR-132 OE
F
exAM microglia
G
significance
FDR < 0.05 & abs(Log2FD) > 0.58
n.s.
DAM
CRM
Homeostatic
Cycling
IRM-like
obs_log2FD
H
Homeostatic
DAM
IRM -like
CRM
Cycling
I
DAM miR-132 OE vs. Ctr
-log(p-value)
logFC
J
GO Biological Process
Protein transport
-log (FDR)
10
1
K
miR-132 KD Pseudotime
miR-132 OE Pseudotime
L
Microglia - Subpopulation
CRM
Cycling
DAM
Homeostatic
IRM
IRM-like
Microglia - Sample
miR-132 KD
Control OE
miR-132 OE
Control KD
Microglia - Experiment
miR-132 KD
miR-132 OE

Figure S3. Characterization of microglial cellular states. Related to Figure 5. A, E. UMAP visualizing subset microglial population from miR-132 KD (A) or miR-132 OE (E) and corresponding control cells. Cells are colored according to identified clusters. UMAPs show integration of samples and different experimental runs, or amount of identified genes, percent of mitochondrial genes and amount of unique molecular identifiers (UMIs) per cell. **B, F.** UMAP visualizing *ex vivo* activated microglial populations from miR-132 KD (B) or miR-132 OE (F) and corresponding control cells. **C, G.** scProportion test comparing miR-132 KD (C) or OE (G) to control cells present in different cellular states. p-value < 0.05 is considered significant. **D, H.** Expression of top 10 enriched genes per cellular state in miR-132 KD (D) or miR-132 OE (H). **I.** Volcano plot showing differentially expressed genes (DEGs) comparing miR-132 OE cells to their corresponding controls in the DAM state. Significant DEGs indicated in red (Wilcoxon rank-sum test using Bonferroni for p-value correction, adjusted p-value < 0.05 considered significant). **J.** GO biological processes significantly enriched among the most differentially expressed genes, with color indicating significance (Fisher's Exact test, p-values corrected with FDR, adjusted p-value < 0.05 considered significant). Homeostatic, homeostatic microglia; DAM, disease-associated microglia; IRM, interferon-response microglia; CRM, cytokine-response microglia; Cycling, cycling microglia. **K.** UMAP of microglial cells ordered along pseudotime. Color indicates pseudotime ordering. **L.** Integrated UMAP of all microglial cells from the miR-132 KD and miR-132 OE experiments. Color indicates the annotated subpopulations (left), sample (middle) or experiment (right).

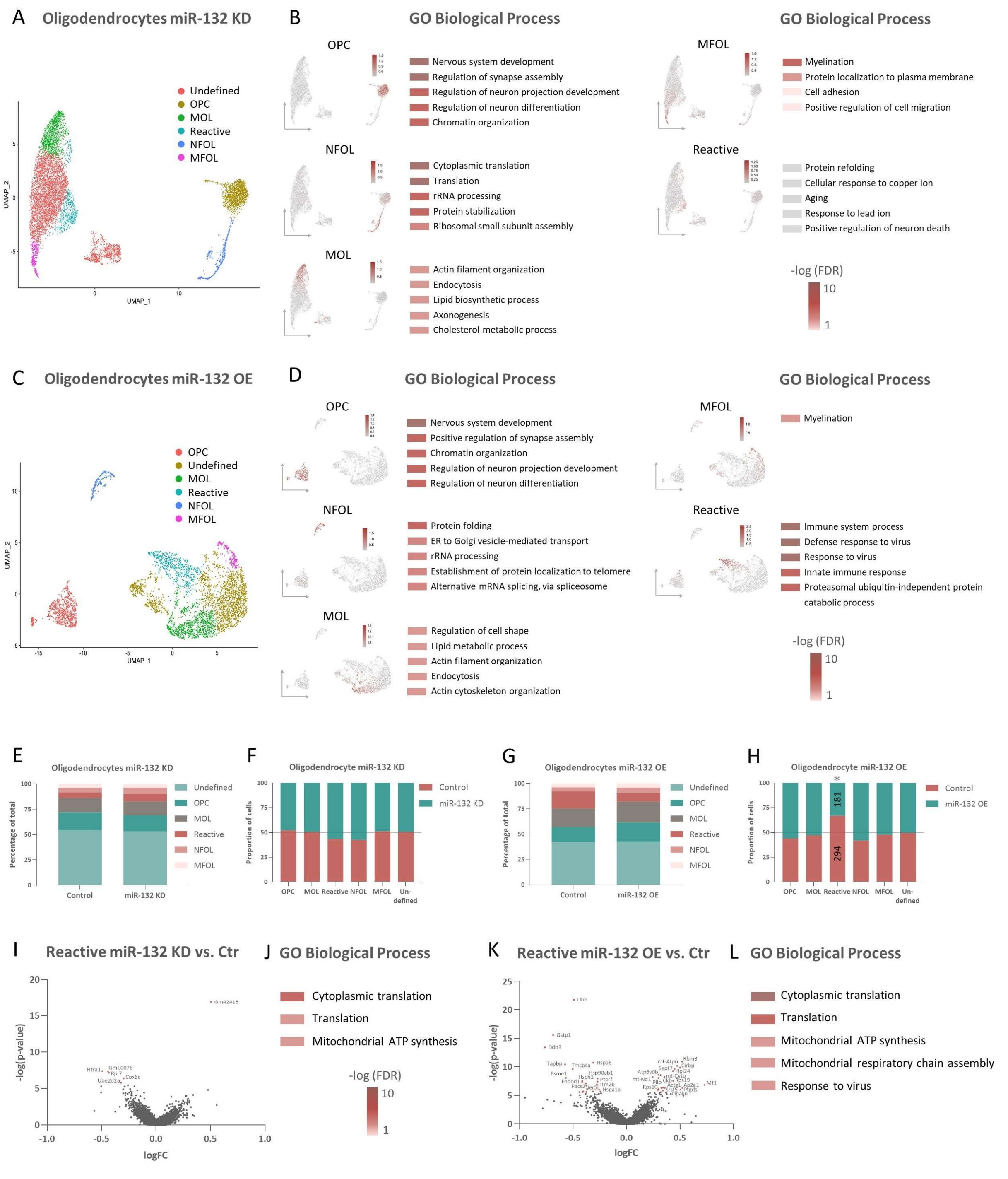

Figure S4. miR-132 overexpression decreases the proportion of reactive oligodendrocytes. Related to Figure S5. A, C. UMAP visualizes subset oligodendrocytic lineage from miR-132 KD (A) or miR-132 OE (C) and corresponding control cells, colored according to identified clusters. **B, D.** UMAP plots colored based on the signature score of the combined gene set that characterizes each individual oligodendrocytic subpopulation. Top 5 unique GO biological processes of significantly enriched genes are provided per subtype; color is indicated according to significance (Fisher's Exact test, p-values corrected with FDR, adjusted p-value < 0.05 considered significant). **E, G.** Normalized percentage of cells present in distinct cellular types/states per condition in miR-132 KD (E) and miR-132 OE (G). **F, H.**

Normalized percentage of cells present in distinct cellular types/states per cell type/state in miR-132 KD (F) and miR-132 OE (H). Significance indicated with an asterisk (Chi-squared test, p-value = 0.0007). The number of cells present in miR-132 OE or control condition is depicted on the graph. **I, K.** Volcano plot showing differentially expressed genes (DEGs) comparing miR-132 KD (I) or miR-132 OE (K) cells to their corresponding controls in reactive oligodendrocytes. Significant DEGs indicated in red (Wilcoxon rank-sum test using Bonferroni for p-value correction, adjusted p-value < 0.05 considered significant). **J, L.** GO biological processes significantly enriched among the top 500 differentially expressed genes (depicted in I, K), with color indicating significance (Fisher's Exact test, p-values corrected with FDR, adjusted p-value < 0.05 considered significant). OPC, oligodendrocyte precursor cell; MOL, mature oligodendrocyte; Reactive, reactive oligodendrocyte; NFOL, newly-formed oligodendrocyte; MFOL, myelin-forming oligodendrocyte.

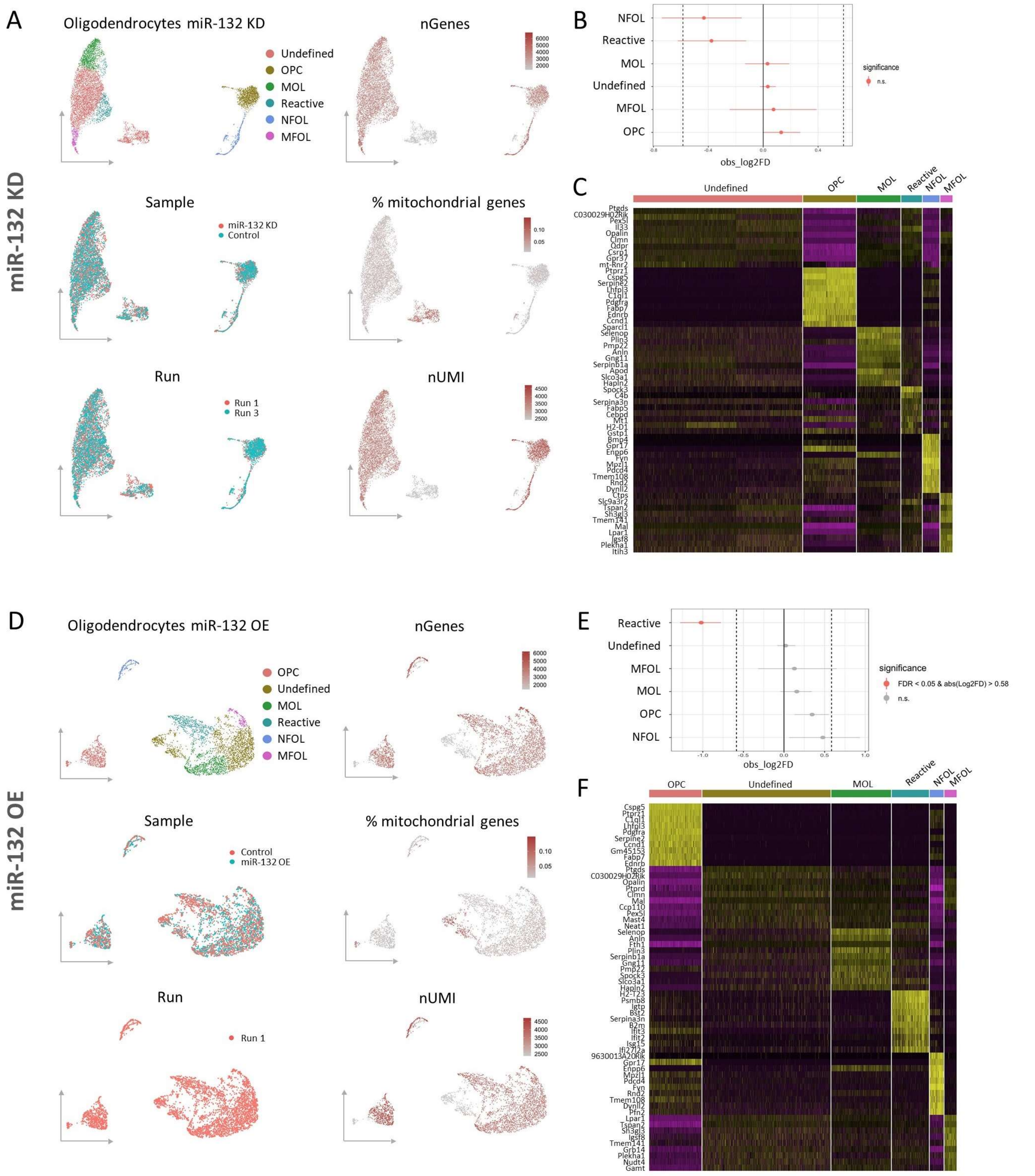

Figure S5. Characterization of oligodendrocytic cellular states. Related to Figure S4. A, D. UMAP visualizing subset oligodendrocytic lineage from miR-132 KD (A) or miR-132 OE (D) and corresponding control cells. Cells are colored according to identified clusters. UMAPs show integration of sample and different experimental runs, or amount of identified genes, percentage of mitochondrial genes or amount of unique molecular identifiers (UMIs) per cell. **B, E.** scProportion test comparing miR-132 KD (B) or OE (E) to control cells present in different cell types. p-value < 0.05 is considered significant. **C, F.** Expression of top 10 enriched genes per cell type in miR-132 KD (C) or miR-132 OE (F). OPC,

oligodendrocyte precursor cell; MOL, mature oligodendrocyte; Reactive, reactive oligodendrocyte; NFOL, newly-formed oligodendrocyte; MFOL, myelin-forming oligodendrocyte.

Table S6: Sequences of primers used. Related to STAR Methods and Figures 6 and S1.

**Primer**	**Sequence**	**Cat#**
mmu-miR-132-3p	UAACAGUCUACAGCCAUGGUCG	204129, Exiqon, Qiagen
**Primer**	**Forward**	**Reverse**
hAPOC1	TGGTTCTGTCGATCGTCTTG	GAAAACCACTCCCGCATCT
hCD9	TTCCTCTTGGTGATATTCGCCA	AGTTCAACGCATAGTGGATGG
hLGALS3	GTGAAGCCCAATGCAAACAGA	AGCGTGGGTTAAAGTGGAAGG
hP2RY12	GAAGACCACCAGGCCATTTA	TCATGCCAGACTAGACCGAAC
hCX3CR1	AGTGTCACCGACATTTACCTCC	AAGGCGGTAGTGAATTTGCAC
hSSH2	TTCAAGGATGGAGGAACTGG	CATGAAGATGTCTGGGCTGA
hPDIA3	AAGCTCAGCAAAGACCCAAA	CACTTAATTCACGGCCACCT
hMAF	AAGTCGACCACCTCAAGCAG	GCTTCCAAAATGTGGCGTAT
hDDX5	GTCAACAAGAGCGTGACTGG	GCGAGCAGTTCTTCCAATTC
hSEC62	AGTAATAGCGGCCACCCTCT	GGAGTCAATGAAGCCCACAT
hCD164	AAGTGGGGAACACGACAGAC	TGAAACTGGCTGCATCAAAG
hUBC	GGGTCGCAGTTCTTGTTTGT	GGAGGGATGCCTTCCTTATC
hPSMB4	TGTCACCGAAAAAGGTGTTG	AAGAGTCTATCTTTGAACTAGCCAAG
hCASP1	AGTGCAGGACAACCCAGCTA	AGATAATGAGAGCAAGACGTGTG
hCASP3	CATGGAAGCGAATCAATGGACT	CTGTACCAGACCGAGATGTCA
hXIAP	GAAGACCCTTGGGAACAACA	TGAAACTGAACCCCATTCGT
hBCL2	GGTATTGGTGAGTCGGATCG	AAGAGTGAGCCCAGCAGAAC
hGAPDH	TCAAGAAGGTGGTGAAGCAGG	ACCAGGAAATGAGCTTGACAAA
mTubb3	TGGAGCGCATCAGCGTATAC	GCCCTGGGCACATACTTGTG
mCd31	AAGCAGCACTCTTGCAGTCA	CATCTCCACGGGTTTCTGTT
mItgam	GGCTTTGGACAGAGTGTGGT	GCTGGGGGACAGTAGAAACA
mAldh1l1	CCATCAACTGGACCCTCATT	CCTGAACCATCCCTTTGATG
mMbp	ACTACCCATTATGGCTCCCTG	GAGGTGGTGTTCGAGGTGTC
mGapdh	TTGATGGCAACAATCTCCAC	CGTCCCGTAGACAAAATGGT
mActin	AGCCATGTACGTAGCCATCC	CTCTCAGCTGTGGTGGTGAA
